# The Stimulator of Interferon Genes Deficiency Attenuates Diabetic Myopathy Through Inhibiting NLRP3‐Mediated Pyroptosis

**DOI:** 10.1002/jcsm.13649

**Published:** 2024-11-27

**Authors:** Jingjuan Yang, Mengqiong Wang, Lingling Shi, Xin Fang, Cui Gao, Lin Ma, Yongfei Wang, Songmin Ying, Yi Yang

**Affiliations:** ^1^ Department of Nephrology, Center for Regeneration and Aging Medicine The Fourth Affiliated Hospital of School of Medicine, and International School of Medicine, International Institutes of Medicine, Zhejiang University, Zhejiang‐Denmark Joint Laboratory of Regeneration and Aging Medicine Yiwu Zhejiang China; ^2^ School of Medicine and Warshel Institute for Computational Biology Chinese University of Hong Kong Shenzhen Guangdong China; ^3^ Department of Paediatrics and Adolescent Medicine University of Hong Kong Hong Kong China; ^4^ Department of Pharmacy, Center for Regeneration and Aging Medicine The Fourth Affiliated Hospital of School of Medicine, and International School of Medicine, International Institutes of Medicine, Zhejiang University, Zhejiang‐Denmark Joint Laboratory of Regeneration and Aging Medicine Yiwu Zhejiang China

**Keywords:** diabetic myopathy, muscle atrophy, muscle dysfunction, NLRP3, pyroptosis, STING

## Abstract

**Background:**

Diabetic myopathy is characterized by the loss of skeletal muscle mass and function. NOD‐like receptor family pyrin domain containing 3 (NLRP3)–mediated pyroptosis is a type of proinflammatory cell death, which can exacerbate significant muscle cell loss and adverse remodelling. The stimulator of interferon genes (STING) is an essential molecule involved in the regulation of inflammation and immune responses across various diseases. The regulatory mechanism by which STING affects muscle pyroptosis in diabetic myopathy remains unclear.

**Methods:**

STING‐knockout and wild‐type (WT) mice underwent intraperitoneal injection of streptozotocin (STZ). STING small interfering RNA (siRNA) was transfected into fully differentiated C2C12 myotubes prior to glucose treatment. Muscle function tests, body composition analysis, transmission electron microscopy, scanning electron microscopy, western blotting, immunofluorescence, immunohistochemistry, histology, enzyme‐linked immunosorbent assay, and reverse transcription polymerase chain reaction were performed. Co‐immunoprecipitation assays were employed to investigate the interaction between STING and NLRP3.

**Results:**

STING expression was elevated in the gastrocnemius muscle (GM) tissues of WT diabetic mice. STING‐deficient diabetic mice exhibited pronounced hyperglycaemia accompanied by hypoinsulinaemia, with no significant difference compared with WT diabetic mice. However, STING‐deficient diabetic mice demonstrated a significantly increased body weight and lean mass. A significant decrease in muscle weight, myofibrillar diameter and area, muscle function, and the expression of genes related to muscle atrophy (MuRF1, Atrogin1) were observed in WT diabetic mice, which was mitigated in STING‐deficient diabetic mice. STING deficiency reduced the number of GSDMD‐N formed pores and pyroptosis‐related components (NLRP3, caspase‐1, cle‐caspase‐1, GSDMD, and GSDMD‐N) in the GM tissues and was associated with a reduction in inflammatory chemokines. Similar changes were observed in vitro with glucose‐induced myotube atrophy and pyroptosis as seen in vivo. Activation of STING by the agonist diABZI exacerbated muscle atrophy and pyroptosis in C2C12 myotubes. Co‐localization of STING and NLRP3 was observed, and the interaction between STING and NLRP3 was enhanced in GM tissues from WT diabetic mice. We also found that STING could activate NLRP3 dependent on its channel activity, which can be attenuated by treated with C53 (an inhibitor of STING's ion‐channel function).

**Conclusions:**

In conclusion, our results indicate that STING‐induced activation of the NLRP3 inflammasome leads to pyroptosis, resulting in muscle atrophy and dysfunction. These findings not only elucidate the mechanism of STING‐induced pyroptosis but also identify STING as a potential therapeutic target for diabetic myopathy.

## 1 Introduction

Type 1 diabetes mellitus (T1D) is characterized by autoimmune‐mediated destruction of pancreatic β cells, resulting in hypoinsulinaemia and hyperglycaemia. T1D is associated with macrovascular and microvascular complications, including nephropathy, retinopathy, and cardiovascular diseases. Insulin therapy remains the primary essential treatment for managing T1D [1]. However, peripheral injections shift insulin action's emphasis to peripheral tissues, particularly skeletal muscle, which is the largest and most metabolically active tissue [2]. Impaired muscle health reduces the muscle's ability to manage fluctuations in glycaemia and lipidaemia, thereby leading to insulin resistance and exacerbating other diabetic complications [2, 3]. Diabetic myopathy, characterized by the loss of skeletal muscle mass and function, can impair quality of life and increase mortality in patients [1]. However, the prognosis and pathophysiological mechanisms of diabetes‐induced myopathy remain inadequately explored.

Pyroptosis is a recently identified form of programmed cell death, characterized by nuclear pyknosis, cell membrane rupture, membrane vesicle formation, and the release of inflammatory cytokines [4]. Pyroptosis is primarily triggered by the activation of the NOD‐like receptor protein 3 (NLRP3) inflammasome and executed by caspase 1 and gasdermin D (GSDMD). The *N*‐terminal domain of GSDMD (GSDMD‐N) causes perforation of the plasma membrane, releasing proinflammatory cytokines such as IL‐1β and IL‐18, which exacerbate the inflammatory cascade [5, 6]. Although pyroptosis was initially observed in macrophages, accumulating evidence indicates that NLRP3 inflammasome–mediated pyroptosis plays a crucial role in diabetes‐associated cardiomyopathy, retinopathy, and myopathy [7, 8, 9, 10]. Targeting pyroptosis has been reported to improve diabetic muscle pathophysiology and muscle function [9]. Therefore, investigating mechanisms that target pyroptosis could offer significant therapeutic potential for diabetic myopathy.

The stimulator of interferon gene (STING) induces type I interferon production by activating the nuclear factor‐κB and interferon regulatory factor 3 transcription pathways [11]. STING plays a critical role in proinflammatory and immunoregulatory processes across various diseases [12, 13, 14]. In diabetic state, the STING signalling pathway is involved in various complications, including diabetic nephropathy and cardiomyopathy [8, 15, 16]. As two central pathways regulating the inflammatory response, STING has been shown to activate the NLRP3 inflammasome in immune cells [17]. However, whether STING also regulates NLRP3 inflammasome–mediated pyroptosis in diabetic myopathy remains unclear, and the intersection between STING and NLRP3 signalling pathways in this condition has not been well elucidated.

This study aimed to investigate whether the STING‐NLRP3 signalling pathway affects skeletal muscle pyroptosis and leads to muscle atrophy and dysfunction in diabetic myopathy. These findings provide a novel mechanism by which STING affects cell pyroptosis and highlight the potential of STING as a therapeutic target for the treatment of diabetic myopathy.

## 2 Methods

### 2.1 Animals

Male C57BL/6J mice aged 6–8 weeks and STING‐deficient mice were obtained from Shanghai SLAC Laboratory and GemPharmatech Co., Ltd., respectively. All mice were housed under specific‐pathogen‐free conditions featuring a 12:12‐h light/dark cycle at a controlled temperature (22°C–24°C) and humidity (50%–60%). Mice were intraperitoneally injected with either sodium citrate (control) or 150 mg/kg streptozotocin (STZ) dissolved in 50‐mM sodium citrate (pH 4.5). One week post‐injection, mice with fasting blood glucose levels exceeding 16.8 mM were classified as diabetes and included in subsequent experiments. Body weight and blood glucose were monitored prior to and weekly after STZ injection. Six weeks post‐STZ injection, muscle function was assessed. Subsequently, the animals were euthanized, and their blood and gastrocnemius muscle samples were collected for further analyses. All experimental procedures received approval from the Zhejiang University Committees for Animal Experiments (Approval Number: ZJU20220235) and adhered to institutional guidelines.

### 2.2 Cell Culture

C2C12 myoblasts and HEK293T cells were maintained in Dulbecco's modified Eagle's medium (DMEM) (glucose 25 mM) supplemented with 10% heat‐inactivated foetal bovine serum (10099141C, Gibco, USA) and 100 units/mL penicillin–streptomycin (C0222, Beyotime, China). Cell lines were cultured at 37°C under a mixture of 95% air and 5% CO_2_. C2C12 myoblasts were passaged before they achieved 80% confluence in order to maintain their undifferentiated state.

The differentiation of C2C12 myoblasts was induced by replacing the growth medium with differentiation medium (DMEM supplemented with 2% horse serum and 100 units/mL penicillin–streptomycin) at 100% confluence. All experiments with C2C12 myotubes were conducted using cells in the fully differentiated state, achieved after 4 days of exposure to differentiation medium. After differentiation, C2C12 myotubes were transferred to DMEM basic medium (glucose 5 mM) for at least 18 h, and subsequently to high‐glucose medium (15, 25, or 30 mM) for the specified durations [18]. High‐glucose medium was prepared by adding d‐(+)‐glucose to DMEM basic medium (glucose 5 mM).

### 2.3 Antibodies and Reagents

The antibodies used were anti‐STING (13647, Cell Signaling Technology, USA), anti‐STING (66680‐1‐Ig, Proteintech, China), anti‐NLRP3 (27458‐1‐AP, Proteintech, China), anti‐GSDMD (AB219800, Abcam, UK), anti‐Caspase1 (AB179515, Abcam, UK), anti‐MuRF1 (55456‐1‐AP, Proteintech, China), anti‐IL18 (60070‐1‐Ig, Proteintech, China), anti‐IL1β (66737‐1‐Ig, Proteintech, China), anti‐GM130 (610822, BD Transduction Laboratories, USA), anti‐MYC tag (16286‐1‐AP, Proteintech, China), anti‐MYC tag (M20002, Abmart, China), anti‐FLAG tag (M20008, Abmart, China), anti‐GAPDH (60004‐1‐Ig, Proteintech, China), anti‐alpha‐Tubulin (66031‐1‐Ig, Proteintech, China), anti‐cGAS (A8335, ABclonal, China), anti‐F4/80 (ab6640, Abcam, UK), anti‐Myosin Heavy Chain (ab37484, Abcam, UK), Cy3 Goat Anti‐Rabbit IgG (H + L) (AS007, ABclonal, China), FITC Goat Anti‐Mouse IgG (H + L) (AS001, ABclonal, China), Alexa Fluor Plus 647 Donkey anti‐Rat IgG (H + L) (A48272, Invitrogen, USA), HRP‐labelled Goat Anti‐Rabbit IgG (H + L) (A0208, Beyotime, China), and HRP‐labelled Goat Anti‐Mouse IgG (H + L) (A0216, Beyotime, China). Antibodies were diluted according to the manufacturer's instructions. The following reagents were used: d‐(+)‐Glucose (G5767, Sigma‐Aldrich, USA), diABZI (tlrl‐diabzi, Invivogen, USA), C53 (37354, Cayman, USA), insulin (HY‐P0035, MedChemExpress, China), and jetPRIME® Versatile DNA/siRNA transfection reagent (101000046, Polyplus, France).

### 2.4 Blood Glucose Levels

Fasting blood glucose levels were measured after 6 h of fasting prior to STZ injection and weekly after STZ injection by FreeStyle Optium Neo Blood Glucose and Ketone Monitoring System (Abbott, Abbott Diabetes Care Ltd., USA).

### 2.5 Body Composition Analysis

Body composition, including the percentages of body mass represented by fat mass (%fat mass) and by lean mass (%lean mass), was measured by using the Nuclear Magnetic Resonance Animal Body Composition Analyzer (QMR06‐090H, NIUMAG, Suzhou, China).

### 2.6 Rotarod Test

To test the mouse muscle function, the rotarod test was measured by using a Panlab LE 8205 rotarod (Panlab Harvard Apparatus, Massachusetts, USA) following the protocol. Briefly, mice were habituated to stay on the spindle for adaptation and training the day before testing. Mice were placed on the rotating lane with an initial speed of 4 rpm and gradually accelerated to 40 rpm over 5 min. Three trials were performed, with a resting period of 20 min between each trial. When the mice dropped safety into its own lane, the latency time to fall (minutes and seconds) and final rotation speed were automatically recorded. The results of three trials were averaged and calculated using latency to fall in seconds. Data were plotted as a bar graph using GraphPad Prism 8 software.

### 2.7 Grip Strength Test

The grip strength test was performed using a grasping force measuring instrument (47200, Ugo Basile, Italy). When the mice grasp a metal grid that is connected to a force transducer, their tails are gently pulled horizontally to produce a force until the grip is released. The results of the grip strength analysis are represented as means of at least three repetitions. Data were plotted as a bar graph using GraphPad Prism 8 software.

### 2.8 RNA Interference (RNAi)

After C2C12 myoblasts differentiation, jetPRIME reagent (2 μL) mixed with siRNA (50 nM) were treated on C2C12 myotubes for 6 h and replaced with fresh medium. After incubation for 24 h after transfection, high glucose medium was treated for 24 h. The siRNAs used in this study were synthesized by GenePharma (Shanghai, China). The corresponding siRNAs used were as follows: m‐si‐STING, sense: 5′‐GCACAUUCGUCAGGAAGAATT‐3′, antisense: 5′‐UAAACCCGAUUCUUGAUGCTT‐3′; m‐si‐cGAS, sense: 5′‐GAACCGGACAAGCUAAAGATT‐3′, antisense:5′‐UCUUUAGCUUGUCCGGUUCTT‐3′; si‐NC, sense: 5′‐UUCUCCGAACGUGUCACGUTT‐3′, antisense: 5′‐ACGUGACACGUUCGGAGAATT‐3′.

### 2.9 Plasmids and Transfection

The STING‐Flag and NLRP3‐Myc plasmids were obtained from RealGene Bio‐Technologies (Shanghai, China). As previously reported, the following STING fragments were subcloned into the pcDNA3.1(+) vector, with the Flag tag oriented at the C‐terminus: those containing certain domain deletions, namely, ΔTM (amino acids 147–379), ΔDD + CBD (amino acids 1–146 + 341–379), and ΔCTT (amino acids 1–340) [19]. The DNA for the full‐length human NLRP3 isoform (amino acids 1–1034) and fragments of the NLRP3 were subcloned into the pCDNA3.1(+) vector, with the Myc tag oriented at the C‐terminus: those containing certain domain deletions, namely, ΔLRR (amino acids 1–697), ΔNACHT (amino acids 1–137 + 698–1034), and ΔPYD (amino acids 138–1034) [20]. Constructs were transfected into the HEK293T cells by transient transfection using jetPRIME® Versatile DNA/siRNA transfection reagent.

### 2.10 RNA Isolation and Real‐Time Quantitative PCR

Total RNA was isolated from GM tissues and C2C12 myotubes using SteadyPure Quick RNA Extraction Kit (AG21023, Accurate Biology, China) according to manufacturer's protocol. cDNA was synthesized using Evo M‐MLV RT Mix Kit with gDNA Clean for qPCR (AG11728, Accurate Biology, China) with 1 μg of RNA. Real‐time quantitative PCR was performed using SYBR Green Premix Pro Taq HS qPCR Kit (AG11701, Accurate Biology, China) on a CFX96 instrument (Bio‐Rad, USA). The expression levels of the target genes were normalized to GAPDH, and fold change values were calculated using the ΔΔCt method. Primer sequence details are list in Table S1.

### 2.11 Enzyme‐Linked Immunosorbent Assay

The mice serum insulin levels at 6‐week post‐STZ injection were measured using commercial Rat/Mouse Insulin ELISA kit (EZRMI‐13K, Millipore, USA) according to the manufacturer's instructions. The concentrations of IL‐1β in the mice serum were measured using commercial mouse IL‐1β ELISA kit (KE10003, Proteintech, China) following the manufacturer's instructions.

### 2.12 Histological Analysis

Pancreas and GM tissues were fixed in 4% paraformaldehyde (PFA), embedded in paraffin, and then sectioned at 4‐μm thickness. The sections were stained with haematoxylin and eosin (H&E) to quantify myofibre area and diameter. C2C12 myoblast differentiation was determined by analysing the myotube area and diameter with H&E staining. The images were obtained under Leica DM4000 microscope (Leica, German). The areas and diameter of muscle fibres were quantified per image with the use of ImageJ software (NIH).

For immunohistochemical analysis, GM sections were deparaffinized and were blocked with H_2_O_2_ (3%) for 20 min. Then the sections were incubated with EDTA/EGTA buffer (pH 9.0) at 100°C for 20 min for antigen retrieval. After subsequent block with 5% bovine serum albumin (BSA) for 1 h at room temperature, the sections were incubated with anti‐STING overnight at 4°C. Staining was performed using the GTVisionTM III Detection System (396 GK500710, Gene Tech, China). The images were obtained under Leica DM4000 microscope (Leica, German).

### 2.13 Immunoblotting Analysis

GM tissues and C2C12 myotubes were lysed by RIPA lysis buffer (P0013B, Beyotime, China) containing 1‐mM PMSF (ST2573, Beyotime, China). The lysates were transferred to 1.5‐mL tube and kept at −20°C before use. Protein samples (20 μg) were loaded onto SDS‐PAGE gels (10%–15%), and then transferred onto 0.22‐μm polyvinylidene difluoride membranes. After being blocked in 5% fat‐free milk in 1X TBST (tri‐buffered saline, 0.1% Tween‐20) at room temperature for 1 h, the blots were incubated with primary antibodies overnight at 4°C and incubated with horseradish peroxidase‐conjugated secondary antibodies. Antigens were visualized using an enhanced chemiluminescence kit (RM00021, ABClonal, China). The protein expression was normalized to endogenous GAPDH. Blot images were obtained using Image Lab software, version 4.1 (Bio‐Rad, USA). Image J software was used to quantify the immunoblotting bands.

### 2.14 Immunofluorescence Staining

For immunofluorescence, PFA‐fixed GM frozen sections or C2C12 myotubes were permeabilized in 0.3% Triton X‐100 for 20 min, blocked in 5% BSA at room temperature for 1 h, and finally incubated with primary antibodies overnight at 4°C. Then the samples were incubated with corresponding secondary antibodies for 1 h at room temperature. The nuclei were counterstained with DAPI. All fluorescence images were captured under the Evident SpinSR confocal microscope (Olympus, Japan).

### 2.15 Co‐Immunoprecipitation Assay

GM tissues and HEK293T cells were collected and lysed on ice in lysis buffer (P0013, Beyotime, China) containing 1‐mM PMSF for 30 min. The lysates were incubated with the indicated primary antibodies or IgG in combination with protein A/G beads (P2108, Beyotime, China) at 4°C overnight under rotary agitation. After 5 washes with 1× TBST, the absorbed proteins were eluted using elution buffer (0.1 M Glycine‐HCl, pH 2.5) followed by SDS‐PAGE and immunoblotting analysis.

#### 2.15.1 Scanning Electron Microscopy

Pyroptotic cell perforation was analysed by scanning electron microscopy. Specimens were washed three times with phosphate buffer, fixed for 120 min in 2% glutaraldehyde and rinsed three times with phosphate buffer. Samples were then fixed for 120 min with osmic acid, rinsed, and dehydrated in a graded series of ethanol concentrations (30%, 50%, 70%, 80%, 90%, 95%, 100%, and 100%) over a period of 120 min and further dehydrated in isoamyl acetate for 15 min. Samples were dropped onto cover slips and dried with a critical point dryer. Specimens are attached to metallic stubs using carbon stickers and sputter coated with gold for 30 s. Finally, images were taken with scanning electron microscopy (SU8100, HITACHI, Japan).

#### 2.15.2 Transmission Electron Microscopy

GM tissues were fixed in glutaraldehyde and osmic acid, dehydrated in ethanol and acetone, postfixed, and dyed with uranyl acetate. After these procedures, the tissues were embedded and polymerized at 37°C overnight. The obtained samples were sectioned to generate ultrathin sections. Next, the sections were sequentially stained with uranyl acetate and lead citrate. The stained tissue sections were imaged using a transmission electron microscope (HT7800, HITACHI, Japan).

### 2.16 Statistics

The data were presented as mean ± standard error of the mean (SEM). Difference was compared using unpaired two‐tailed Student's *t* test between two groups or two‐way analysis of variance among more than two groups. All statistical analyses were performed using GraphPad software 8.0. A *p* value < 0.05 was considered statistically significant.

#### 2.16.1 Role of the Funders

This work was supported by grants from Zhejiang Provincial Nature Science Foundation of China (No. LZ24H050001) and the National Nature Science Foundation of China (82170681). These funders were not involved in any aspects regarding conduction, analysis, interpretation, or publication of the study.

## 3 Results

### 3.1 STING Expression Was Elevated in GM Tissues and STING Deficiency Prevented Skeletal Muscle Decline in STZ‐Induced Diabetic Mice

**FIGURE 1:**
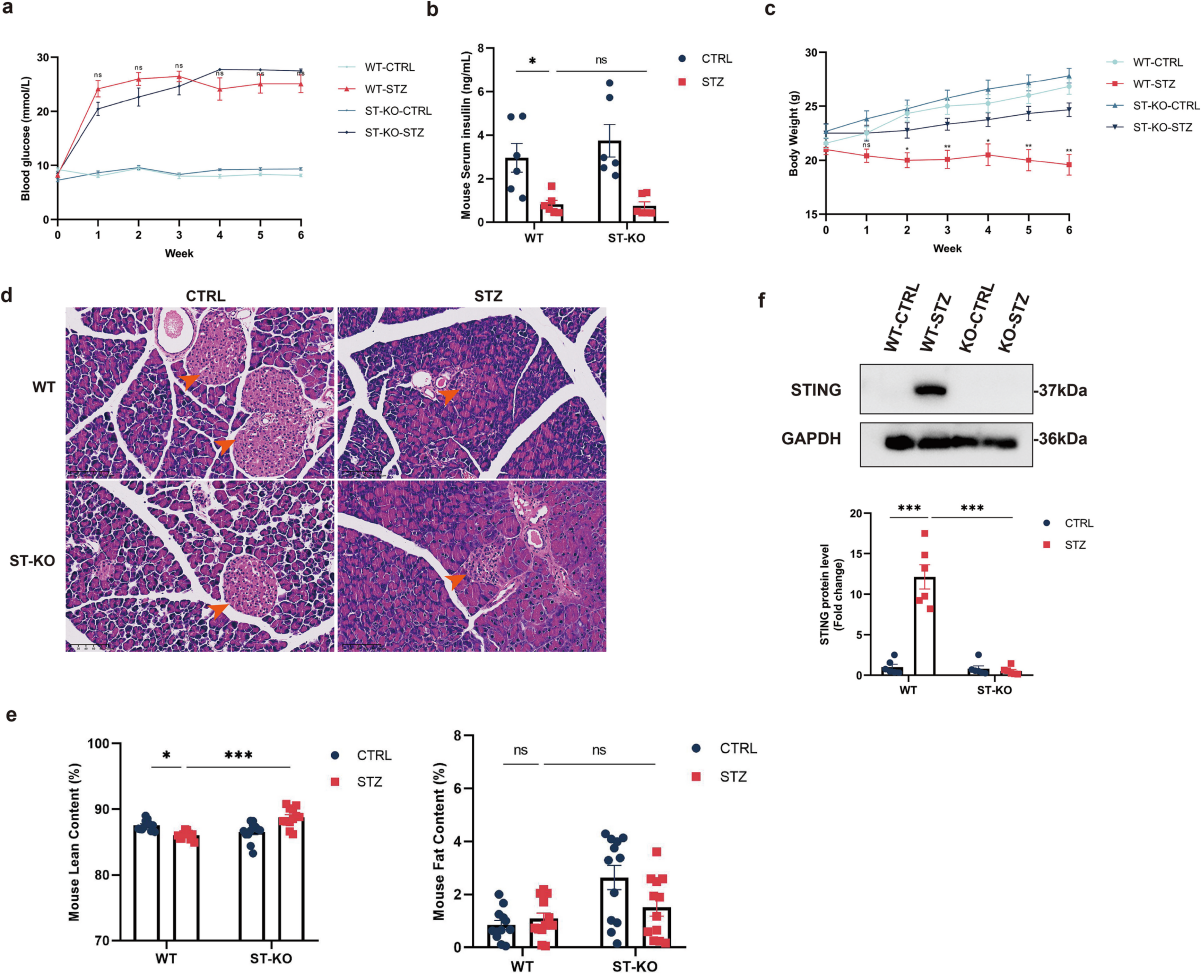
STING expression was elevated in GM tissues and STING deficiency prevented skeletal muscle decline in STZ‐induced diabetic mice. Wild‐type (WT) and STING‐deficient mice were administered 150 mg/kg STZ to induce the diabetes model. STZ‐induced diabetes was confirmed by elevated glucose levels (a, ns vs. ST‐KO‐STZ group) and hypoinsulinaemia after 6 weeks of STZ administration (b) (*n* = 6). (c) Body weight was measured weekly following STZ administration; ns, *p < 0.05, **p < 0.01, ***p < 0.001 versus ST‐KO‐STZ group (*n* = 6). (d) Haematoxylin and eosin (H&E) staining of pancreatic tissue showed a significant decrease in the number and volume of islets. The orange arrows indicate islets. Scale bars, 100 μm. (e) Lean and fat content were measured after 6 weeks of STZ administration using body composition analysis (*n* = 12). (f) Western blot analysis of STING expression levels in GM tissues of WT and STING‐KO mice (*n* = 6). Data were analysed using an unpaired two‐tailed Student's *t* test for comparisons between two groups, or two‐way analysis of variance for more than two groups. Data are presented as mean ± SEM. *p < 0.05, **p < 0.01, *p < 0.001; ns, not significant. GM, gastrocnemius muscle; CTRL, control; STZ, streptozotocin; WT, wild‐type; ST‐KO, STING‐KO.

To elucidate the mechanism by which diabetes mellitus promotes skeletal muscle decline, a mouse model of STZ‐induced diabetes was constructed. Intraperitoneal injection of STZ in WT mice led to marked hyperglycaemia (Figure 1a), accompanied by hypoinsulinaemia (Figure 1b) and a loss of body mass (Figure 1c). Haematoxylin and eosin (H&E) staining of pancreas tissue revealed a significant reduction in both the number and volume of islets in diabetic mice (Figure 1d). Compared with control, body composition analysis indicated a significant decrease in lean mass in WT diabetic mice, with no significant differences in fat mass (Figure 1e). The expression level of STING in GM tissues from STZ‐induced diabetic mice was then assessed. As shown in Figure 1f, the protein expression level of STING was significantly elevated in GM tissues of WT diabetic mice. Consistently, immunohistochemical staining further confirmed the elevated STING expression in GM tissues of WT diabetic mice (Figure S1). These findings indicate that STING is elevated in GM tissues of STZ‐induced diabetic mice.

To investigate the role of STING in diabetes‐induced skeletal muscle decline, global STING‐deficient mice were used in this study. STING protein expression was absent in GM tissues from STING‐deficient mice (Figure 1f; Figure S1). Following intraperitoneal injection of STZ, STING‐deficient mice also exhibited marked hyperglycaemia (Figure 1a), accompanied by hypoinsulinaemia (Figure 1b), with no significant difference compared with WT diabetic mice. However, compared with WT diabetic mice, STING‐deficient diabetic mice demonstrated increased body mass (Figure 1c). Additionally, body composition analysis indicated a significant increase in lean mass in STING‐deficient diabetic mice, with no significant differences in fat mass (Figure 1e). These results indicate that compared with WT diabetic mice, STING‐deficient diabetic mice increased body weight primarily by gaining muscle mass without affect glucose metabolism.

### 3.2 STING Deficiency Attenuated Muscle Atrophy and Dysfunction in STZ‐Induced Diabetic Mice

**FIGURE 2:**
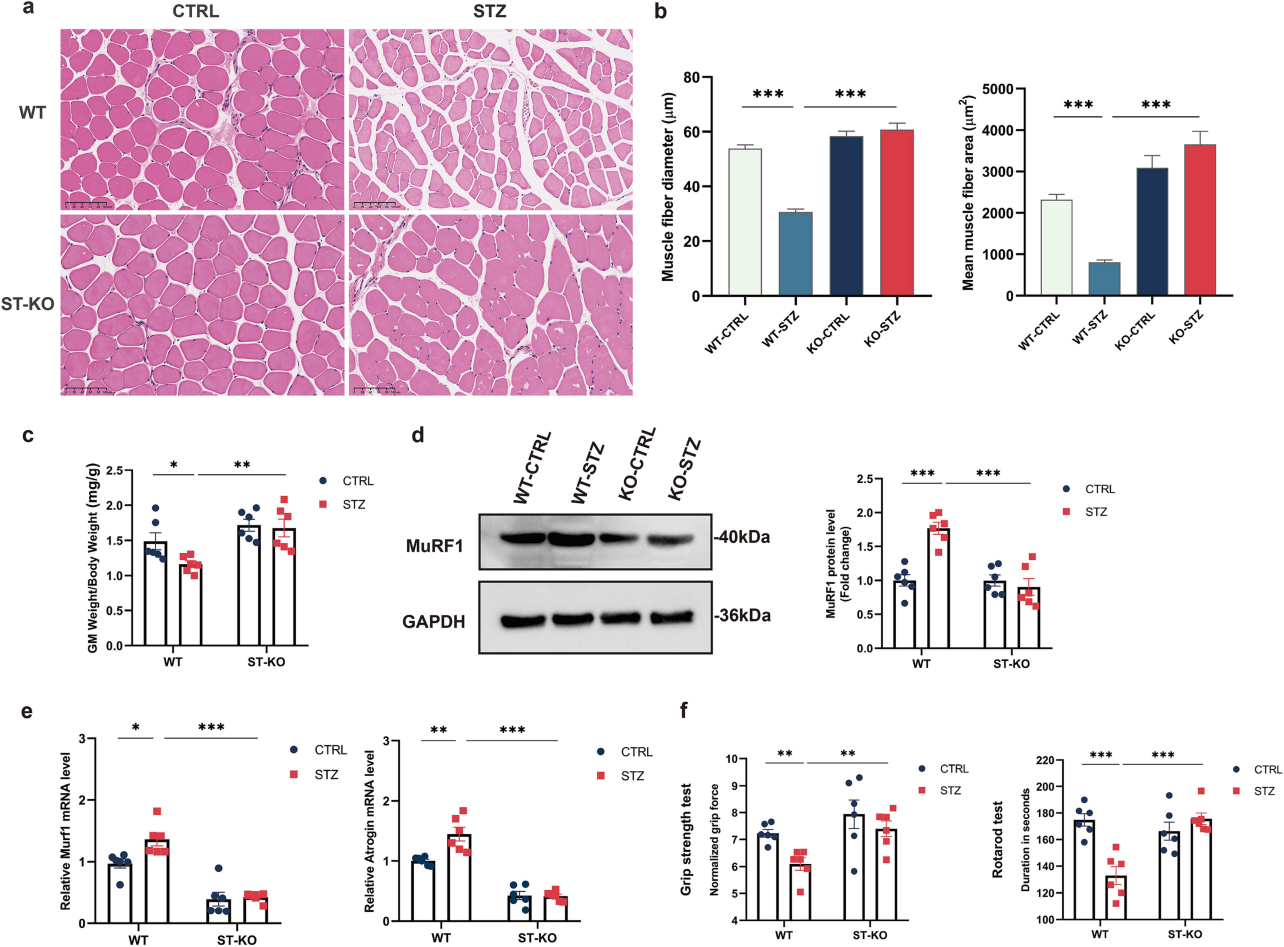
STING deficiency attenuated muscle atrophy and dysfunction in STZ‐induced diabetic mice. WT or STING KO mice were studied at 6 weeks following STZ administration or vehicle injection. (a) Gastrocnemius muscle (GM) morphology was examined through haematoxylin and eosin (H&E) staining. Scale bars, 100 μm. (b) Analysis of the area and diameter of GM fibres was conducted. Muscle fibre area and diameter were calculated using ImageJ software. Bars represent the mean ± SEM. (c)The ratio of muscle mass to body mass was measured in WT and STING KO mice (*n* = 6). (d) Immunoblotting analysis was performed to assess MuRF1 protein levels in GM tissues of WT and STING KO mice. Quantification of MuRF1 protein levels was conducted (*n* = 6). (e) Quantitative analysis of mRNA expression of atrophy‐related genes in GM tissues (*n* = 6). At 6 weeks following STZ administration, animals underwent various muscle function tests. (f) Quantification and analysis of the four‐limb grip strength test and latency to fall in the rotarod test were performed for WT and STING KO mice (*n* = 6). Data were analysed using two‐way analysis of variance and are presented as mean ± SEM. *p < 0.05, **p < 0.01, *p < 0.001. GM, gastrocnemius muscle; CTRL, control; STZ, streptozotocin; WT, wild‐type; ST‐KO, STING‐KO.

As STING‐deficient diabetic mice increase body weight, we questioned the role of STING in muscle atrophy and dysfunction. To evaluate the effect of STING on muscle atrophy, we conducted H&E staining of GM tissues, measured the fibre diameter and area, calculated the ratio of GM‐to‐body weight, and assessed the expression of genes related to muscle atrophy. As shown in Figure 2a, compared with control mice, representative photomicrographs demonstrated a significant reduction in GM fibre size, suggesting muscle atrophy in WT diabetic mice. A significant increase in GM fibre size was shown in STING‐deficient diabetic mice, suggesting alleviation in muscle atrophy. Quantification of the H&E staining demonstrated that the muscle fibre area and diameter size were significantly increased in STING‐deficient diabetic mice compared with WT diabetic mice (Figure 2b). Our results showed a significant decrease in GM mass of WT mice following STZ treatment as compared with control, which was attenuated in STING‐deficient diabetic mice (Figure 2c). The protein expression level of MuRF1, a key regulator of muscle atrophy [21], was significantly elevated in STZ‐induced mice compared with control, and the STZ‐induced increase was inhibited in STING‐deficient mice (Figure 2d). Also, the mRNA expression of genes related to muscle atrophy, Murf1 and Atrogin1, was increased in WT mice by STZ treatment, which was also alleviated in STING‐deficient diabetic mice (Figure 2e).

To investigate the effect of STING on the muscle function, mice were subjected to two different types of tests: grip strength test and rotarod test. The grip strength test revealed a significant decrease in grip strength in WT diabetic mice as compared with the controls, whereas grip strength was significantly improved in STING‐deficient diabetic mice (Figure 2f). Additionally, in the rotarod test, WT diabetic mice demonstrated significantly reduced endurance at maximum speed and quicker falls, indicating decreased muscle function; these impairments were prevented in STING‐deficient diabetic mice (Figure 2f). Collectively, these results indicate that STING deficiency attenuates muscle atrophy and dysfunction in STZ‐induced diabetic mice.

### 3.3 STING Deficiency Inhibited the NLRP3 Inflammasome–Mediated Pyroptosis in Gastrocnemius Muscle of STZ‐Induced Diabetic Mice

**FIGURE 3:**
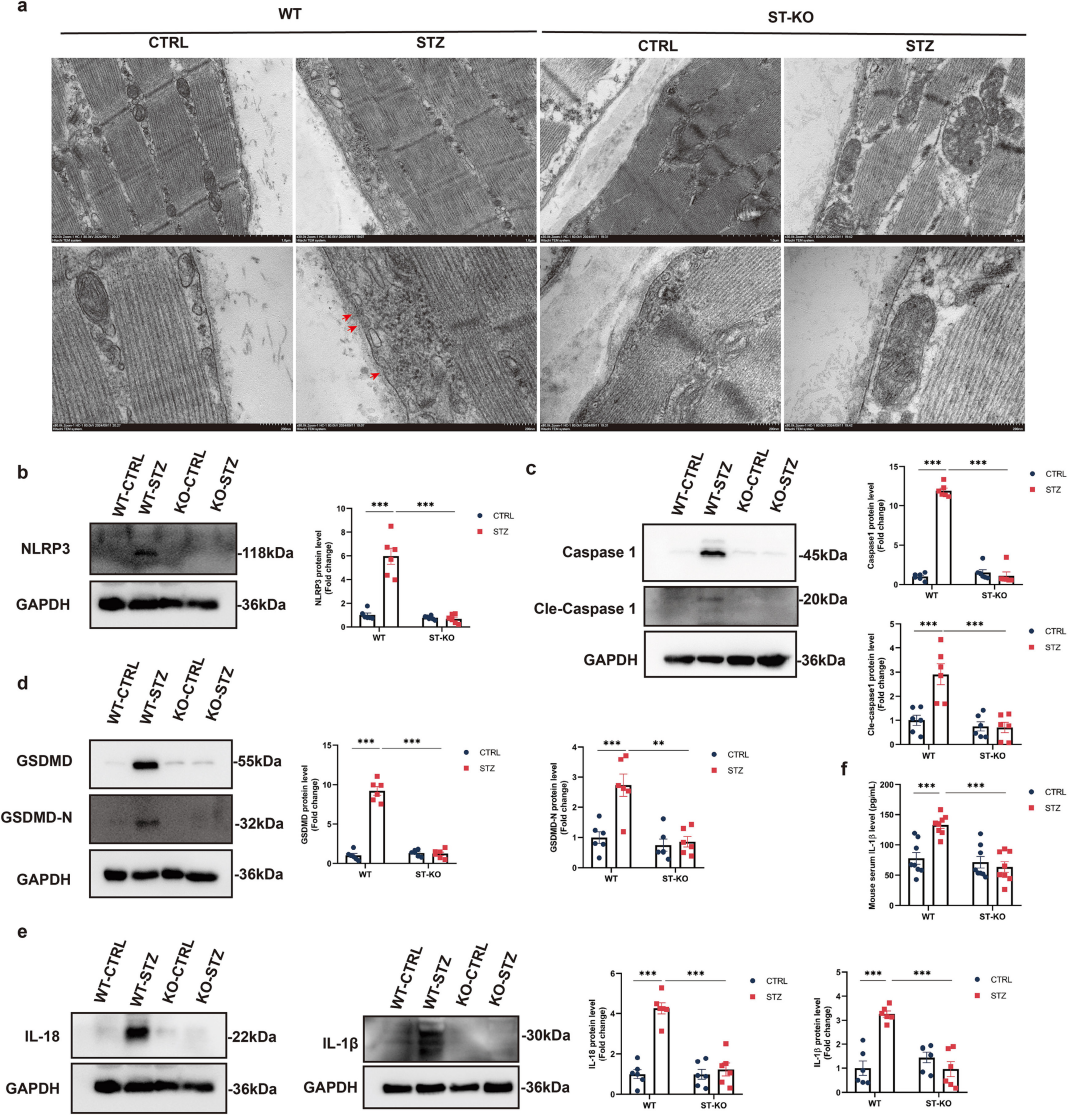
STING deficiency inhibited the NLRP3 inflammasome and pyroptosis in gastrocnemius muscle of STZ‐induced diabetes mice. (a) Representative transmission electron micrographs of gastrocnemius muscle (GM) tissues. The bottom panel shows the inset of the upper panel at higher magnification. Red arrows indicate pyroptosis pores on the plasma membrane. (b‐e) Western blot analysis of the expression levels of STING, NLRP3 (b), Caspase‐1 and cle‐Caspase‐1 (c), GSDMD and GSDMD‐N (d), IL‐1β and IL‐18 (e) in GM tissues of wild‐type (WT) and STING‐knockout (KO) mice (*n* = 6). (f) Quantitative ELISA analysis of IL‐1β levels in the serum of WT and STING KO mice (*n* = 6). Data were analysed using two‐way analysis of variance and are presented as mean ± SEM. *p < 0.05, **p < 0.01, *p < 0.001. GM, gastrocnemius muscle; CTRL, control; STZ, streptozotocin; WT, wild‐type; ST‐KO, STING‐KO.

The NLRP3 inflammasome–mediated pyroptosis plays a critical role in diabetic myopathy [9, 22]. Recent studies have demonstrated that targeting pyroptosis can ameliorate muscle pathophysiology and function in diabetes [9, 22]. Transmission electron microscopy revealed a reduced number of GSDMD‐N formed pores in the GM tissues of STING‐deficient diabetic mice compared with WT diabetic mice (Figure 3a). To elucidate the role of STING in NLRP3 inflammasome–mediated pyroptosis in diabetic myopathy, the protein levels of pyroptosis‐related components, including NLRP3, caspase‐1, cleaved caspase‐1 (cle‐caspase‐1), GSDMD, and GSDMD‐N, were assessed. The results indicated significant increases in the protein levels of NLRP3, caspase‐1, cle‐caspase‐1, GSDMD, and GSDMD‐N in GM tissues from WT diabetic mice. Additionally, the STZ‐induced increases in pyroptosis‐related molecules were abolished in STING‐deficient diabetic mice (Figure 3b–d).

To assess the expression of pyroptosis‐related pro‐inflammatory cytokines, IL‐1β and IL‐18 levels were measured in GM tissue. The protein levels of IL‐1β and IL‐18 were significantly elevated in WT diabetic mice and decreased in STING‐deficient diabetic mice (Figure 3e). Additionally, the mRNA levels of IL‐1β and IL‐18 were significantly upregulated in WT diabetic mice and attenuated by STING deficiency (Figure S2). As shown in Figure 3f, serum IL‐1β levels were significantly increased in WT diabetic mice, and this increase was reversed in STING‐deficient diabetic mice. These results suggest that STING deficiency inhibited the NLRP3 inflammasome and pyroptosis in the gastrocnemius muscle of STZ‐induced diabetic mice.

### 3.4 STING Deficiency Alleviated Muscle Atrophy and NLRP3 Inflammasome–Mediated Pyroptosis in Glucose‐Treated Mouse C2C12 Myotubes

**FIGURE 4:**
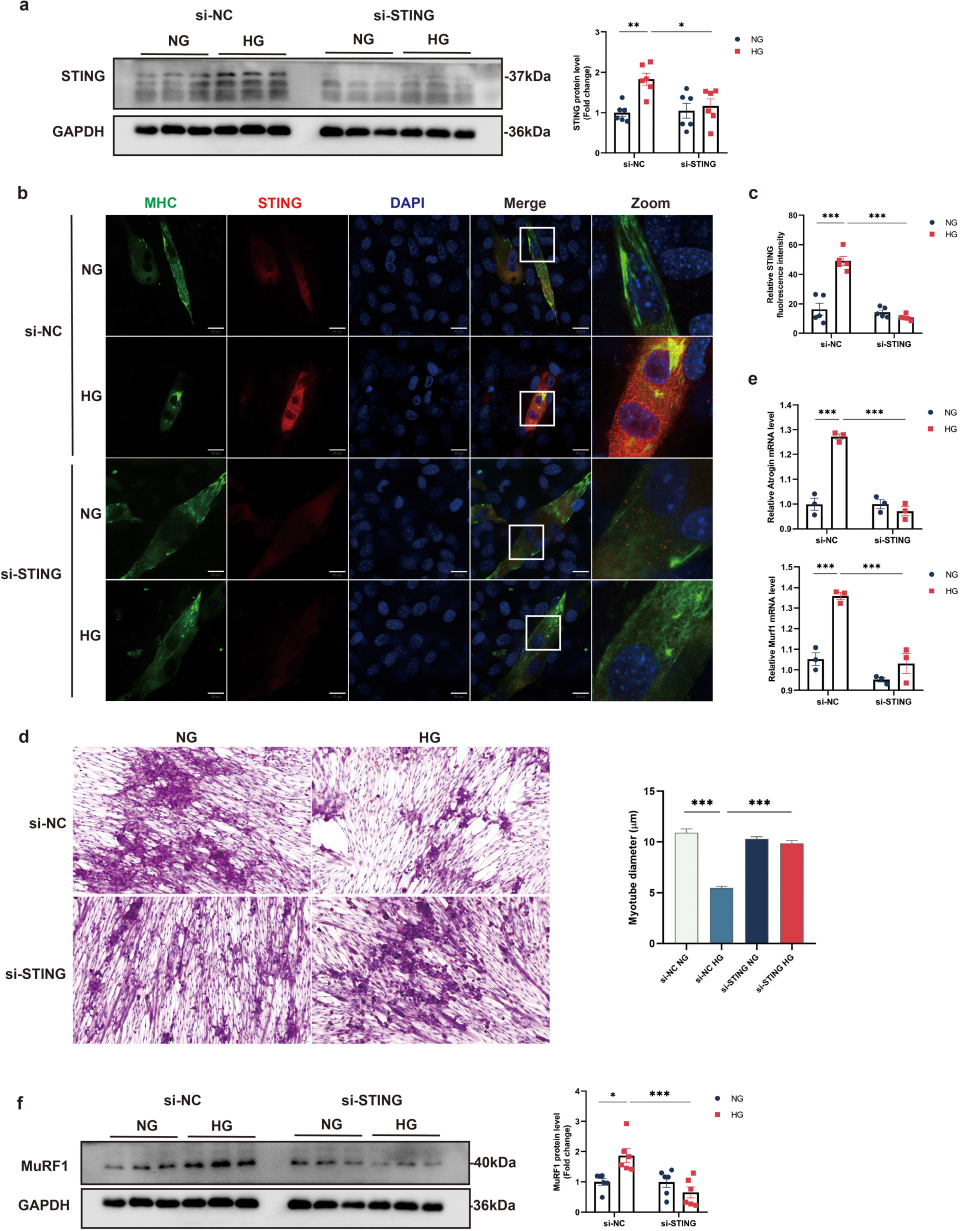
STING deficiency alleviated muscle atrophy in glucose‐treated mouse C2C12 myotubes. (a) Immunoblotting analysis of STING in C2C12 myotubes, with quantification of STING protein levels (*n* = 6). (b) C2C12 myotubes treated with glucose were subjected to immunofluorescence analysis using anti‐MHC (green), anti‐STING (red), DAPI (blue), and confocal microscopy. The rightmost panel shows the inset of the left panel at higher magnification. Scale bar, 20 μm. (c) Quantification of STING fluorescence intensity (*n* = 5). (d) C2C12 myotube morphology was examined through haematoxylin and eosin (H&E) staining. Scale bar, 50 μm. Myotube diameter was calculated using ImageJ software. (e) Quantitative analysis of mRNA expression of atrophy‐related genes in C2C12 myotubes (*n* = 3). (f) Immunoblotting analysis of MuRF1 in C2C12 myotubes was performed, with quantification of MuRF1 protein levels (*n* = 6). Data were analysed using two‐way analysis of variance and are presented as mean ± SEM. *p < 0.05, **p < 0.01, *p < 0.001. NG, normal glucose; HG, high glucose.

Both hyperglycaemia and hypoinsulinaemia are present in STZ‐induced diabetes. Exposure of C2C12 myotubes to glucose increased STING protein levels in a concentration‐dependent manner (Figure S3). In contrast, treatment with insulin had no effect on STING protein levels in C2C12 myotubes (Figure S4), suggesting that hyperglycaemia directly leads to the elevation of STING protein in the skeletal muscle of diabetic mice. Thus, a glucose concentration of 30 mM was used in subsequent cell experiments to induce hyperglycaemia. Glucose exposure led to increased STING protein levels in C2C12 myotubes (Figure 4a). Immunofluorescent staining further confirmed the elevated STING expression in C2C12 myotubes (Figure 4b–c). Morphological analysis revealed that glucose exposure caused atrophy of C2C12 myotubes and significantly reduced myotube diameter (Figure 4d). The mRNA expression levels of muscle atrophy‐related genes, Atrogin1 and Murf1, were also significantly increased in glucose‐treated C2C12 myotubes (Figure 4e). To further assess STING's role in muscle atrophy, si‐STING was used to treat C2C12 myotubes, which were then exposed to high glucose. si‐STING transfection significantly downregulated STING, as determined by immunoblotting (Figure 4a). Morphological analysis showed that si‐STING treatment alleviated myotube atrophy, as evidenced by increased myotube diameter (Figure 4c). Meanwhile, MuRF1 protein expression levels were significantly elevated in glucose‐treated C2C12 myotubes, and this increase was prevented in si‐STING transfected myotubes (Figure 4f).

**FIGURE 5:**
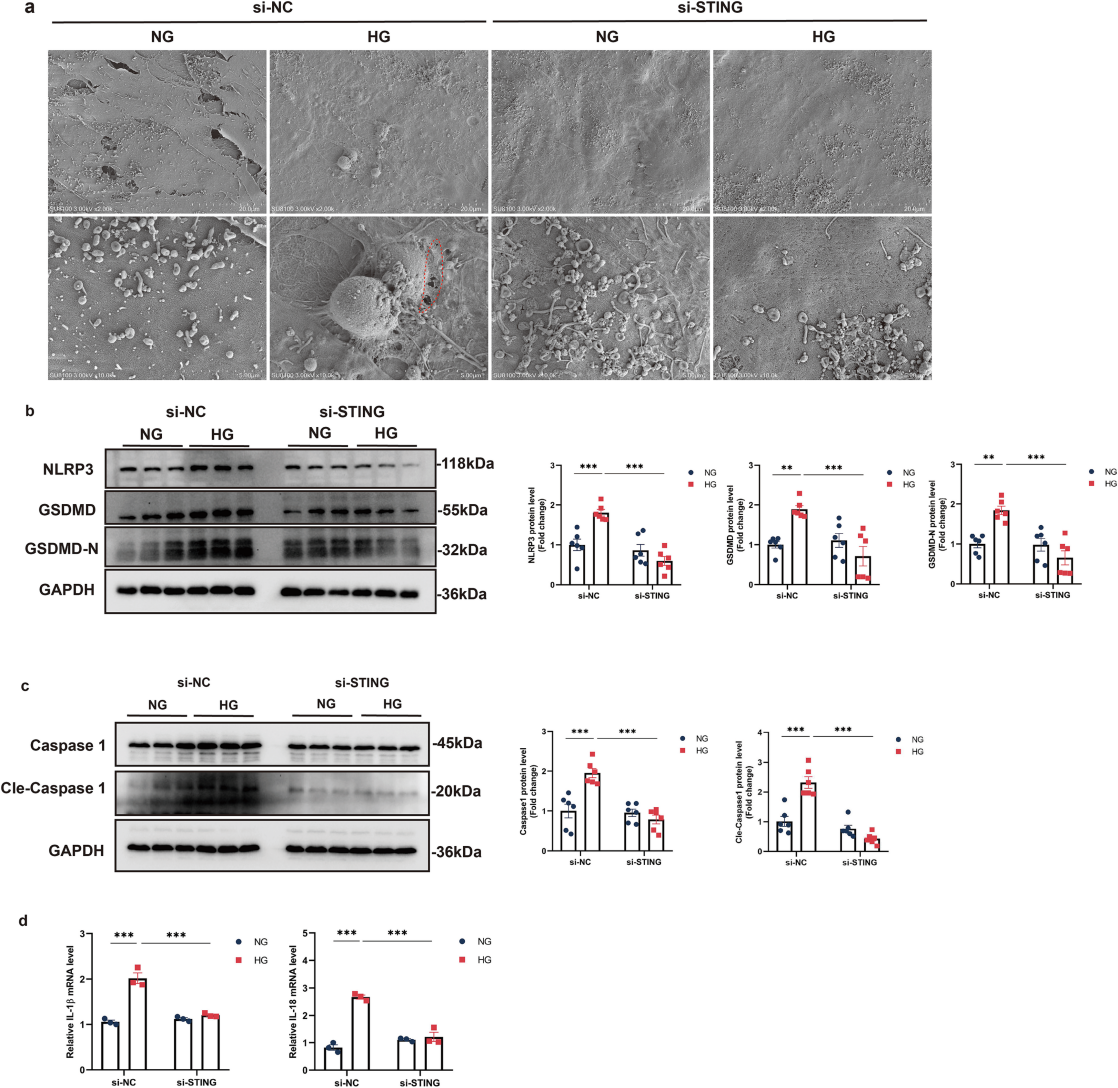
STING deficiency alleviated pyroptosis in glucose‐treated mouse C2C12 myotubes. (a) Representative scanning electron micrographs of C2C12 myotubes. The bottom panel shows the inset of the upper panel at higher magnification. Red arrows indicate pyroptosis pores. (b‐c) Western blot analysis of the expression levels of NLRP3 (b), GSDMD, GSDMD‐N (b), Caspase‐1, and cle‐Caspase‐1 (c) in C2C12 myotubes (*n* = 6). (d) Quantitative analysis of mRNA expression of IL‐1β and IL‐18 in C2C12 myotubes (*n* = 3). Data were analysed using two‐way analysis of variance and are presented as mean ± SEM. *p < 0.05, **p < 0.01, *p < 0.001. NG, normal glucose; HG, high glucose.

Glucose treatment in C2C12 myotubes resulted in the formation of GSDMD‐N pores, a typical feature of pyroptotic cell morphology, compared with controls (Figure 5a). Additionally, protein levels of pyroptosis components, including NLRP3, caspase‐1, cle‐caspase‐1, GSDMD, and GSDMD‐N, were significantly increased following glucose treatment. However, the pyroptotic cell morphology and increased levels of pyroptosis components were significantly reversed in si‐STING transfected myotubes (Figure 5a–c). Additionally, IL‐1β and IL‐18 expression levels were significantly elevated in glucose‐exposed C2C12 myotubes, and these increases were reversed in si‐STING transfected myotubes (Figure 5d). Collectively, these results indicate that STING deficiency alleviates muscle atrophy and pyroptosis in glucose‐treated mouse C2C12 myotubes.

### 3.5 Activating STING Contributed to Muscle Atrophy and Pyroptosis in Mouse C2C12 Myotubes

**FIGURE 6:**
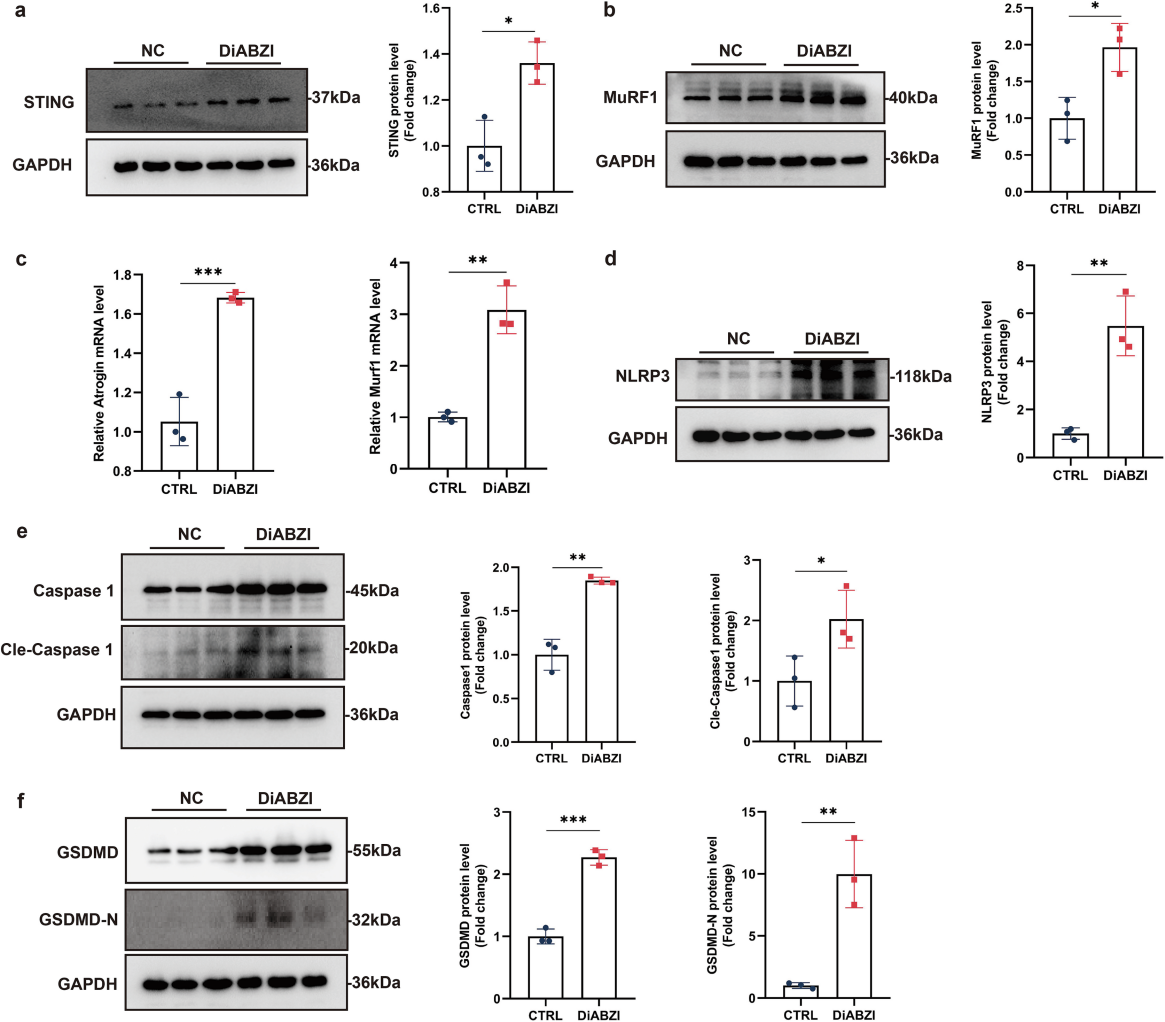
Activating STING contributed to muscle atrophy and pyroptosis in mouse C2C12 myotubes. (a) Immunoblotting analysis of STING in C2C12 myotubes, with quantification of STING protein levels (*n* = 3). (b) Immunoblotting analysis of MuRF1 in C2C12 myotubes, with quantification of MuRF1 protein levels (*n* = 3). (c) Quantitative analysis of mRNA expression levels of atrophy‐related genes in C2C12 myotubes (*n* = 3). (d‐f) Western blot analysis of the expression levels of NLRP3 (d), Caspase‐1, cle‐Caspase‐1 (e), GSDMD, and GSDMD‐N (f) in C2C12 myotubes (*n* = 3). Data were analysed using an unpaired two‐tailed Student's *t* test and are presented as mean ± SEM. *p < 0.05, **p < 0.01, *p < 0.001.

To further elucidate the impact of STING activation on muscle atrophy and pyroptosis, mouse C2C12 myotubes were treated with diABZI, a STING agonist, at a concentration of 10 μM. DiABZI is known to be internalized into the cytoplasm via an unknown receptor, leading to STING activation [23]. Following a 24‐h treatment with diABZI, an increase in STING expression was observed (Figure 6a). This treatment also induced muscle atrophy, as evidenced by the upregulation of Atrogin1 and Murf1 (Figure 6b,c).

We then examined the pyroptotic response in mouse C2C12 myotubes induced by diABZI. The results indicated that the protein levels of NLRP3, caspase 1, cle‐caspase 1, GSDMD, and GSDMD‐N were significantly increased in the C2C12 myotubes after diABZI treatment (Figure 6d–f). Additionally, the expression of IL‐1β and IL‐18 was significantly elevated in the mouse C2C12 myotubes following diABZI treatment (Figure S5). Taken together, the activation of STING by the agonist diABZI also led to muscle atrophy and pyroptosis in mouse C2C12 myotubes.

### 3.6 Glucose Exposure Mediated the Binding of STING to NLRP3

**FIGURE 7:**
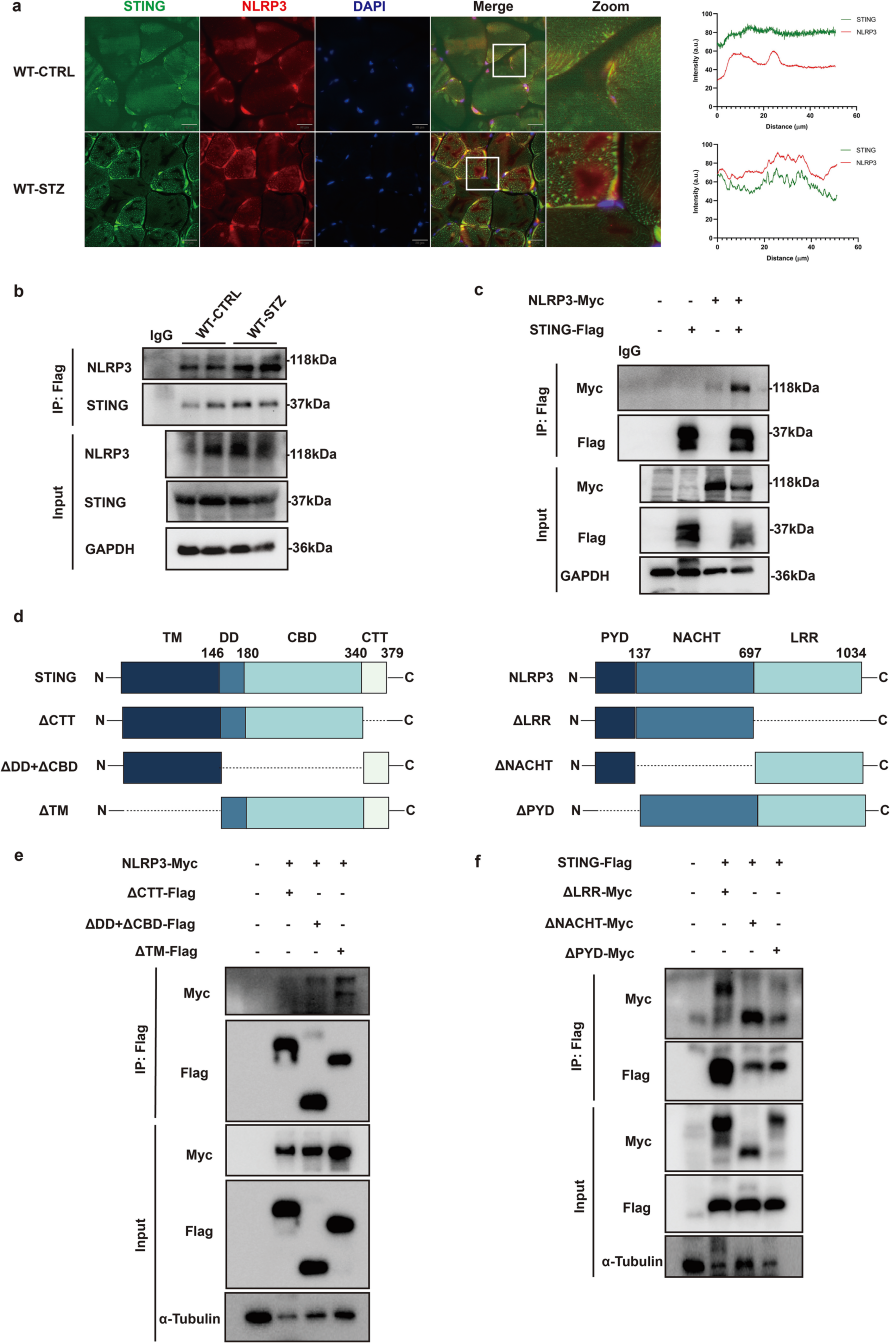
Glucose exposure mediated the binding of STING to NLRP3. (a) Representative confocal images showing co‐localization of STING and NLRP3 in the gastrocnemius muscle (GM) tissues of wild‐type (WT) mice. Scale bar, 20 μm. Co‐localization analysis was performed using ImageJ software. (b) Co‐immunoprecipitation showing the interaction between STING and NLRP3 in GM tissues of WT mice. (c) Co‐immunoprecipitation of STING‐Flag with NLRP3‐Myc in HEK293T cells. (d) Schematic structure of STING and NLRP3 proteins. (e) Flag immunoprecipitation (IP) from lysates of HEK293T cells overexpressing Flag‐tagged STING fragments and Myc‐tagged NLRP3 was followed by immunoblotting. (f) Flag IP from lysates of HEK293T cells overexpressing Myc‐tagged NLRP3 fragments and Flag‐tagged STING was followed by immunoblotting. GM, gastrocnemius muscle; STZ, streptozotocin; WT, wild‐type; ST‐KO, STING knockout (STING‐KO).

The STING‐NLRP3 pathway is critical in regulating inflammation. As shown in Figure 7a, there existed co‐localization of STING and NLRP3 in the GM tissues of WT diabetic mice. Co‐immunoprecipitation experiments conducted on GM tissues from WT diabetic mice explored the endogenous association between STING and NLRP3. Western blot analysis confirmed that the interaction between STING and NLRP3 was enhanced in GM tissues of WT diabetic mice (Figure 7b). Additionally, co‐transfection of HEK293T cells with STING‐Flag and NLRP3‐Myc plasmids revealed that STING can interact with NLRP3 (Figure 7c).

To identify which domains of STING and NLRP3 were responsible for their interaction, we constructed three STING truncated mutants: Flag‐ΔTM, Flag‐ΔDD + CBD, and Flag‐ΔCTT (Figure 7d). We co‐transfected truncated STING proteins with Myc‐tagged full‐length (FL) NLRP3 in HEK293T cells. Co‐immunoprecipitation experiments with these truncated mutants revealed that Flag‐ΔCTT lost the ability to interact with NLRP3, whereas Flag‐ΔTM and Flag‐ΔDD + CBD did not (Figure 7e). These findings suggested that NLRP3 interacts with STING at the CTT domain. In addition, we constructed three NLRP3 truncated mutants:Myc‐ΔLRR, Myc‐ΔNACHT, and Myc‐ΔPYD, which were co‐transfected with FL‐Flag‐STING in HEK293T cells (Figure 7d). The results showed that the PYD of NLRP3 was the main domain involved in the STING interaction (Figure 7f).

### 3.7 STING Activated NLRP3 Inflammasome Dependent on STING's Channel Activity and Induced NLRP3 Translocation to the Golgi Apparatus

**FIGURE 8:**
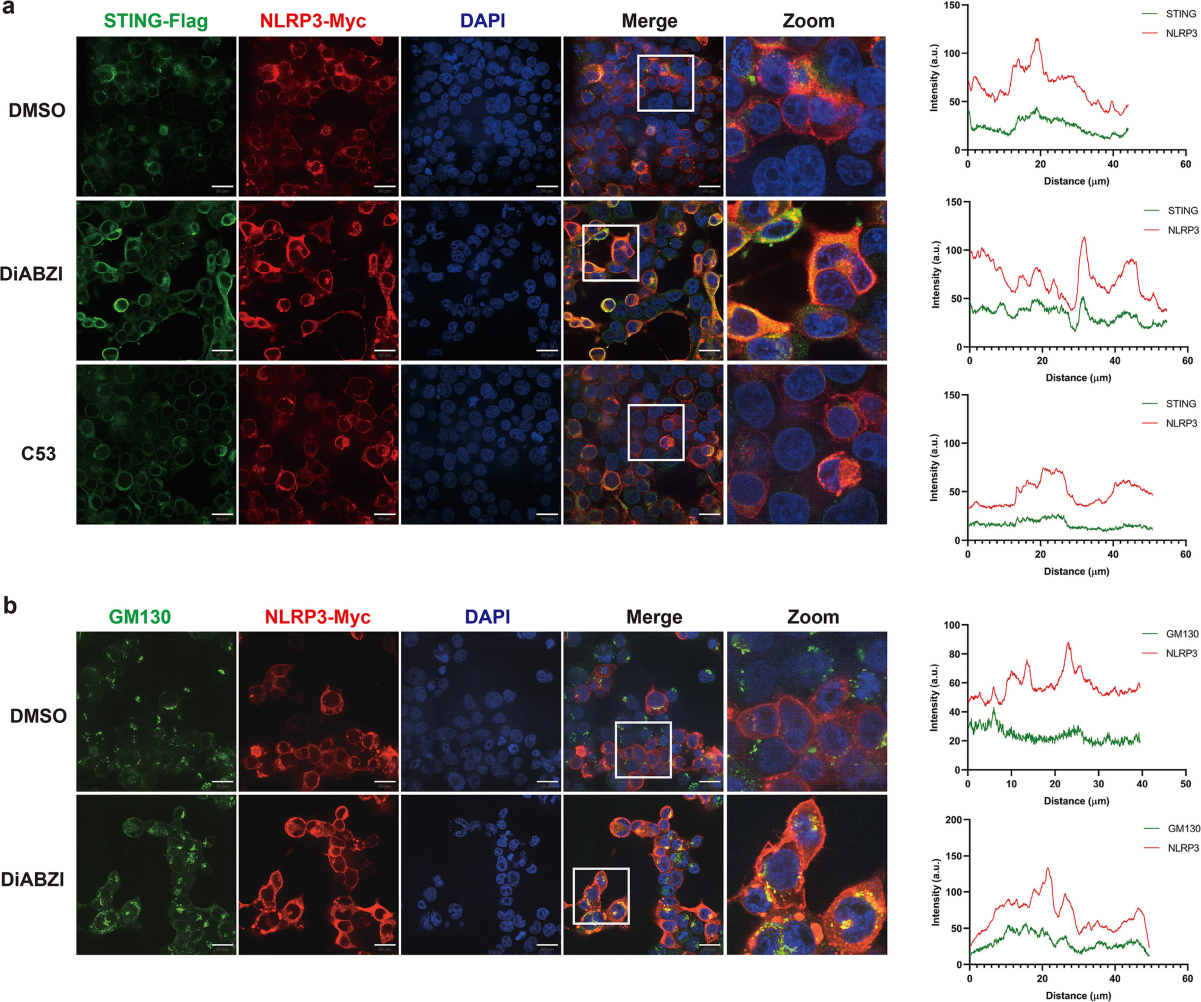
STING activated NLRP3 inflammasome dependent on STING's channel activity and induced NLRP3 translocation to the Golgi apparatus. (a) Representative images of STING and NLRP3 in HEK293T cells expressing STING‐Flag and NLRP3‐Myc treated with DMSO, 1‐μM diABZI, with or without 10‐mM C53 for 1 h. Scale bar, 20 μm. Quantification of STING and NLRP3 co‐localization in HEK293T cells expressing STING‐Flag and NLRP3‐Myc. (b) Representative images of NLRP3 and GM130 (a Golgi marker) in HEK293T cells expressing STING‐Flag and NLRP3‐Myc treated with DMSO and 1‐μM diABZI for 6 h. Quantification of NLRP3 and GM130 co‐localization in HEK293T cells expressing STING‐Flag and NLRP3‐Myc. Co‐localization analysis was performed using ImageJ software.

Recent studies have demonstrated that STING can activate the NLRP3 inflammasome by inducing proton leakage, similar to the activity of the influenza M2 protein. Upon activation, NLRP3 translocate from the cytosol to Golgi vesicles, initiating downstream inflammasome activation [24]. To explore whether STING can activate the NLRP3 inflammasome by inducing proton leakage, HEK293T cells were co‐transfected with STING‐Flag and NLRP3‐Myc plasmids. It was observed that NLRP3 formed puncta upon stimulation with the STING agonist diABZI (Figure 8a). Consistent with the mechanism by which STING activates the NLRP3 inflammasome through proton leakage induction, a significant reduction in NLRP3 translocation was observed when cells were treated with both diABZI and C53 (an inhibitor of STING's ion‐channel function) (Figure 8a) [25]. This suggests that STING activates the NLRP3 inflammasome by inducing proton leakage.

As demonstrated in Figure 8a, NLRP3 co‐localized with STING in these puncta. STING can activate the NLRP3 inflammasome through proton leakage at the Golgi, dependent on its channel activity. It was found that NLRP3 co‐localized with GM130, a Golgi marker, upon diABZI stimulation (Figure 8b). Collectively, these results indicate that STING activation of the NLRP3 inflammasome depends on its channel activity, facilitating NLRP3 translocation to the Golgi apparatus.

## 4 Discussion

Herein, it was reported that STING expression was upregulated in GM tissues of diabetic mice. STING could activate NLRP3 inflammasome–mediated pyroptosis, ultimately leading to skeletal muscle atrophy and dysfunction. In addition, we identified STING could bind to NLRP3, which provided a basis for a physical interaction between these effectors. It was also found that STING could activate NLRP3 dependent on its channel activity, and induce NLRP3 translocation to the Golgi apparatus, thereby promoting pyroptosis. These findings not only elucidate the mechanism of STING‐induced pyroptosis but also reveal STING as a potential therapeutic target for diabetic myopathy.

Previous studies have analysed the ultrastructure of skeletal muscle in T1D patients and observed reductions in myofibre and myofibril diameters [26, 27]. In nondiabetic states, skeletal muscle can regulate its intracellular glucose concentration; however, in T1D patients, repeated hyperinsulinaemia from recurrent peripheral insulin injections leads to substantially increased glucose flux into muscles, ultimately resulting in repeated intracellular hyperglycaemia [2, 28].Increased expression of STING has been reported in various diabetic organs, including nephropathy [15], cardiomyopathy [8], wound [29], and retinopathy [30]. The present study also found elevated STING levels in the skeletal muscles of diabetic mice, and STING knockout did not affect glucose and insulin metabolism. STING, a multifunctional protein, is involved not only in the secretion of type I IFNs but also in the regulation of inflammation [31]. Recent research has demonstrated that increased hyperglycaemia in both diabetic patients and animal models is associated with heightened levels of inflammation [32, 33]. Therefore, it is proposed that STING activation may regulate diabetic myopathy through the mediation of inflammation in skeletal muscle.

STING is a conserved mammalian cytoplasmic receptor that plays a crucial role in infection, inflammation, and immunity‐related conditions [34, 35]. Aberrant dsDNA accumulation, under stress conditions or pathogen infections, is detected by cyclic GMP‐AMP (cGAMP) synthase (cGAS), which catalyses the production of cGAMP. Upon binding to cGAMP, STING undergoes a conformational change and translocates from the endoplasmic reticulum (ER) to the Golgi apparatus, where it mediates several biological functions, including interferon induction, noncanonical light‐chain 3B (LC3B) lipidation, and NLRP3 inflammasome activation [17, 36]. To assess whether cGAS could activate STING in diabetic myopathy, si‐cGAS was used to incubate with C2C12 myotubes. Following glucose treatment, si‐cGAS transfection significantly downregulated STING expression, as determined by immunoblotting (Figure S6). Our data indicated that cGAS lead to STING activation in diabetic myopathy. However, the factors influencing the cGAS‐STING axis and other specific mechanisms of STING activation still warrant further investigation.

Pyroptosis, a form of proinflammatory cell death, has been identified in various cell types across numerous diseases [37]. Recent studies have demonstrated that NLRP3 inflammasome activation significantly contributes to inflammation and pyroptosis, leading to myocyte dysfunction and cell death, ultimately resulting in muscle atrophy [38, 39]. Nora et al. revealed that NLRP3 inflammasome activation is a key cause of inflammation, leading to muscle atrophy in wild‐type and NLRP3 knockout mice with septicaemia [38]. Gao et al. demonstrated that inhibiting NLRP3/GSDMD‐mediated pyroptosis attenuates skeletal muscle atrophy [39]. In diabetic states, elevated hyperglycaemia and inflammation induce cellular pyroptosis, resulting in substantial muscle cell loss and adverse remodelling in diabetic myopathy [9]. Consistent with previous findings, our data demonstrated that STING deficiency alleviated NLRP3‐mediated pyroptosis and reduced muscle atrophy and dysfunction. As macrophages are essential for skeletal muscle homeostasis, and pyroptosis was originally observed in macrophages, immunofluorescence was used to assess whether STING regulates macrophage pyroptosis in GM tissues. As shown in Figure S7, STING deficient did not affect macrophage pyroptosis in GM tissues. However, whether macrophage pyroptosis plays a role in the regulation of diabetic myopathy requires further investigation.

Of note, the mechanism by which STING regulates pyroptosis is complex and involves numerous molecular pathways. Recent study revealed that STING activates the phosphorylation and nuclear translocation of IRF3, subsequently inducing NLRP3‐mediated pyroptosis in LPS‐treated cardiomyocytes [13]. Additionally, in LPS‐induced acute lung injury, STING activation by cytoplasmic mtDNA and c‐Myc can promote NLRP3 inflammasome and macrophage pyroptosis [12]. STING also activates pyroptosis via interactions with the NLRP3 inflammasome in microglia during ischemic stroke [6]. Hacohen et al. discovered that STING functions as a proton channel, inducing a pH increase in the Golgi and activating the NLRP3 inflammasome via proton leakage [24]. NLRP3 activation involves its translocation from the cytosol to Golgi vesicles, subsequently inducing downstream inflammasome activation [40]. In our study, we found that STING activated the NLRP3 inflammasome via proton leakage, as NLRP3 translocation was significantly reduced when cells were treated with diABZI and C53. Additionally, we identified that STING could bind to NLRP3, providing a basis for a physical interaction between these effectors. These findings suggest that STING likely regulates NLRP3 activation, either directly or indirectly.

This study had several limitations. We evaluated the effects of STING on pyroptosis using global knockout mice; however, mice with conditional STING knockout in GM tissues are required to validate the current findings. Second, we did not investigate the role of insulin therapy in STING‐NLRP3 signalling and pyroptosis. However, treatment of C2C12 myotubes with insulin had no effect on STING expression. Future studies should investigate the role of insulin therapy on STING‐NLRP3 signalling and pyroptosis.

In conclusion, our findings demonstrate that STING is significantly upregulated in GM tissues of diabetic mice, and STING deficiency alleviates pyroptosis, muscle atrophy, and dysfunction. Furthermore, STING‐induced activation of the NLRP3 inflammasome leads to pyroptosis, resulting in muscle atrophy and dysfunction (Figure S8). These findings not only shed light on the mechanism of STING‐induced pyroptosis but also suggest STING as a potential therapeutic target for diabetic myopathy.

## Conflicts of Interest

The authors declare no conflict of interest. Figure S8 is created using Figdraw (www.figdraw.com).

## Supporting information


**Figure S1.** **Expression of STING in GM tissues.** Representative immunohistochemical images of STING staining and H&E staining. Scale bars, 100 μm. GM, gastrocnemius muscle; STZ, streptozotocin; WT, wild type; ST‐KO, STING‐KO.
**Figure S2.** **The mRNA levels of IL‐1β and IL‐18 in GM tissues of STZ‐induced diabetes mice.** Data were analysed using two‐way analysis of variance. Data are presented as mean ± SEM. *p < 0.05, **p < 0.01. GM, gastrocnemius muscle; STZ, streptozotocin; WT, wild type; ST‐KO, STING‐KO.
**Figure S3.** **Exposure of C2C12 myotubes to glucose increased the protein level of STING in a concentration‐dependent manner**. Data were analysed using unpaired two‐tailed Student’s t‐test. Data are presented as mean ± SEM. *p < 0.05, **p < 0.01.
**Figure S4.** **Treatment of the C2C12 myotubes with insulin had no effect on the protein level of STING**. Data were analysed using unpaired two‐tailed Student’s t‐test. Data are presented as mean ± SEM; ns, not significant.
**Figure S5.** **Activating STING contributed to muscle atrophy and pyroptosis in mouse C2C12 myotubes**. Quantitative PCR analysis of mRNA levels of IL‐1β and IL‐18 (*n* = 3). **Data were analysed using unpaired two‐tailed Student’s t‐test.** Data are presented as mean ± SEM. *p < 0.05, ***p < 0.001.
**Figure S6.** **Transfected with si‐cGAS significantly downregulated STING expression in mouse C2C12 myotubes treated with glucose.** Data were analysed using unpaired two‐tailed Student’s t‐test. Data are presented as mean ± SEM; **p < 0.01.
**Figure S7.** **STING deficient did not affect the macrophage pyroptosis in GM tissues.** Representative images of GSDMD and F4/80 (macrophage marker) in GM tissues. Scale bar, 20 μm. GM, gastrocnemius muscle; STZ, streptozotocin; WT, wild type; ST‐KO, STING‐KO.
**Figure S8.** **A mechanism diagram**. STING‐induced activation of the NLRP3 inflammasome could lead to pyroptosis, resulting in muscle atrophy and dysfunction in diabetes. ER, endoplasmic reticulum.
**Figure S9.** **Original blots for western blot assay in the present study.**
**Table S1.** **Primers used for qRT‐PCR**.
