# Supplementary material for: The Stimulator of Interferon Genes Deficiency Attenuates Diabetic Myopathy Through Inhibiting NLRP3‐Mediated Pyroptosis
Source: J Cachexia Sarcopenia Muscle. 2024 Nov 27;16(1):e13649. doi: 10.1002/jcsm.13649 (PMC11670168; doi:10.1002/jcsm.13649)
Supplement: Supplementary file 1 — Figure S1. Expression of STING in GM tissues. Representative immunohistochemical images of STING staining and H&E staining. Scale bars, 100 μm. GM, gastrocnemius muscle; STZ, streptozotocin; WT, wild type; ST‐KO, STING‐KO. Figure S2. The mRNA levels of IL‐1β and IL‐18 in GM tissues of STZ‐induced diabetes mice. Data were analysed using two‐way analysis of variance. Data are presented as mean ± SEM. *p < 0.05, **p < 0.01. GM, gastrocnemius muscle; STZ, streptozotocin; WT, wild type; ST‐KO, STING‐KO. Figure S3. Exposure of C2C12 myotubes to glucose increased the protein level of STING in a concentration‐dependent manner. Data were analysed using unpaired two‐tailed Student’s t‐test. Data are presented as mean ± SEM. *p < 0.05, **p < 0.01. Figure S4. Treatment of the C2C12 myotubes with insulin had no effect on the protein level of STING. Data were analysed using unpaired two‐tailed Student’s t‐test. Data are presented as mean ± SEM; ns, not significant. Figure S5. Activating STING contributed to muscle atrophy and pyroptosis in mouse C2C12 myotubes. Quantitative PCR analysis of mRNA levels of IL‐1β and IL‐18 (n = 3). Data were analysed using unpaired two‐tailed Student’s t‐test. Data are presented as mean ± SEM. *p < 0.05, ***p < 0.001. Figure S6. Transfected with si‐cGAS significantly downregulated STING expression in mouse C2C12 myotubes treated with glucose. Data were analysed using unpaired two‐tailed Student’s t‐test. Data are presented as mean ± SEM; **p < 0.01. Figure S7. STING deficient did not affect the macrophage pyroptosis in GM tissues. Representative images of GSDMD and F4/80 (macrophage marker) in GM tissues. Scale bar, 20 μm. GM, gastrocnemius muscle; STZ, streptozotocin; WT, wild type; ST‐KO, STING‐KO. Figure S8. A mechanism diagram. STING‐induced activation of the NLRP3 inflammasome could lead to pyroptosis, resulting in muscle atrophy and dysfunction in diabetes. ER, endoplasmic reticulum. Figure S9. Original blots for western blot assay in the pres [file JCSM-16-e13649-s001.docx]

**Supplementary figures and tables**

**The Stimulator of Interferon Genes Deficiency Attenuates Diabetic Myopathy through Inhibiting NLRP3-mediated Pyroptosis**

Jingjuan Yang^1^, Mengqiong Wang^1^, Lingling Shi^1^, Xin Fang^1^, Cui Gao^1^, Lin Ma^1^, Yongfei Wang^2,3^, Songmin Ying^4, **^, Yi Yang^1, *^

^1^Department of Nephrology, Center for Regeneration and Aging Medicine, the Fourth Affiliated Hospital of School of Medicine, and International School of Medicine, International Institutes of Medicine, Zhejiang University, Zhejiang-Denmark Joint Laboratory of Regeneration and Aging Medicine, Yiwu, Zhejiang, China

^2^School of Medicine and Warshel Institute for Computational Biology, Chinese University of Hong Kong, Shenzhen, Guangdong, China

^3^Department of Paediatrics and Adolescent Medicine, University of Hong Kong, Hong Kong, China

^4^Department of Pharmacy, Center for Regeneration and Aging Medicine, the Fourth Affiliated Hospital of School of Medicine, and International School of Medicine, International Institutes of Medicine, Zhejiang University, Zhejiang-Denmark Joint Laboratory of Regeneration and Aging Medicine, Yiwu Zhejiang, China

Songmin Ying: yings@zju.edu.cn; Yi Yang: yangyixk@zju.edu.cn

^*^Corresponding author. Department of Nephrology, Center for Regeneration and Aging Medicine, the Fourth Affiliated Hospital of School of Medicine, and International School of Medicine, International Institutes of Medicine, Zhejiang University, Zhejiang-Denmark Joint Laboratory of Regeneration and Aging Medicine, Yiwu, Zhejiang, China. [yangyixk@zju.edu.cn](mailto:yangyixk@zju.edu.cn)

^**^Corresponding author. Department of Pharmacy, Center for Regeneration and Aging Medicine, the Fourth Affiliated Hospital of School of Medicine, and International School of Medicine, International Institutes of Medicine, Zhejiang University, Zhejiang-Denmark Joint Laboratory of Regeneration and Aging Medicine, Yiwu Zhejiang, China. yings@zju.edu.cn

**
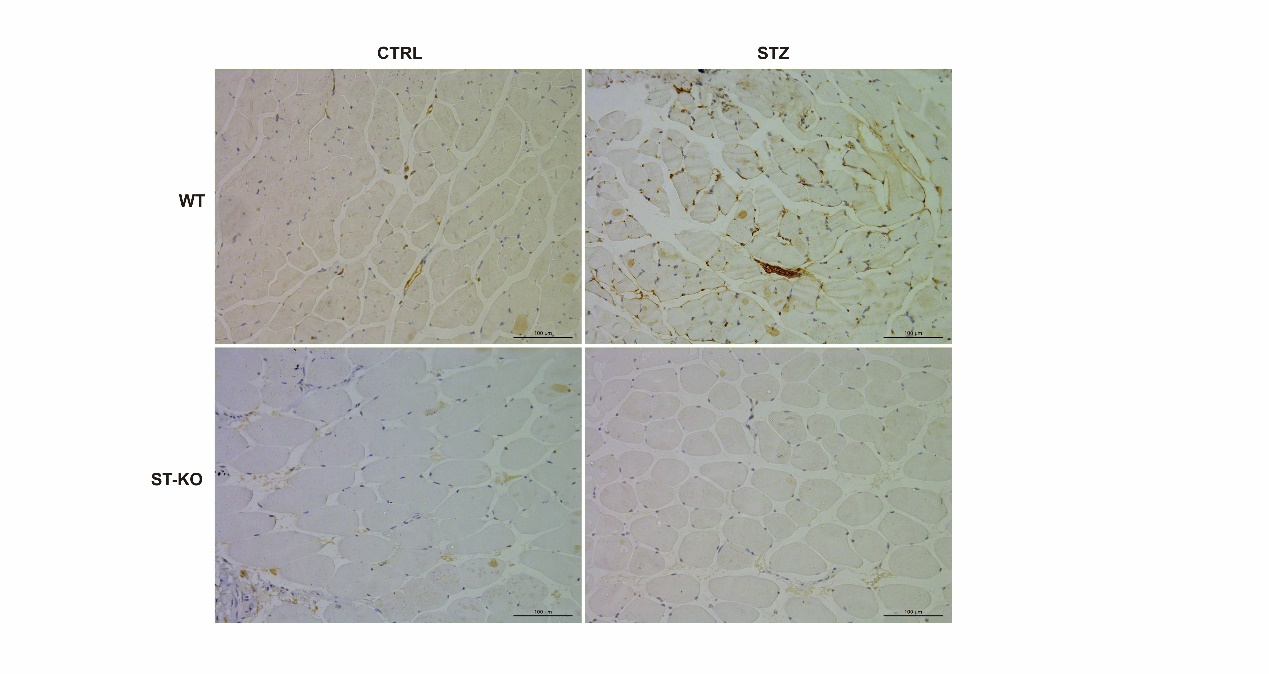
**

**Figure S1. Expression of STING in GM tissues.** Representative immunohistochemical images of STING staining and H&E staining. Scale bars, 100 μm. GM, gastrocnemius muscle; STZ, streptozotocin; WT, wild type; ST-KO, STING-KO.

**
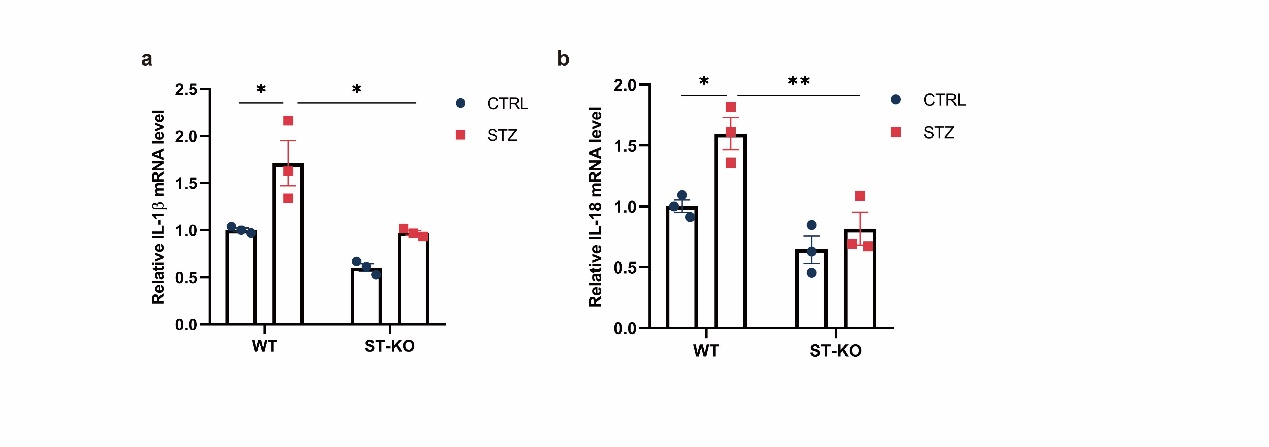
**

**Figure S2.** **The mRNA levels of IL-1β and IL-18 in GM tissues of STZ-induced diabetes mice.** Data were analyzed using two-way analysis of variance. Data are presented as mean ± SEM. *p<0.05, **p<0.01. GM, gastrocnemius muscle; STZ, streptozotocin; WT, wild type; ST-KO, STING-KO.


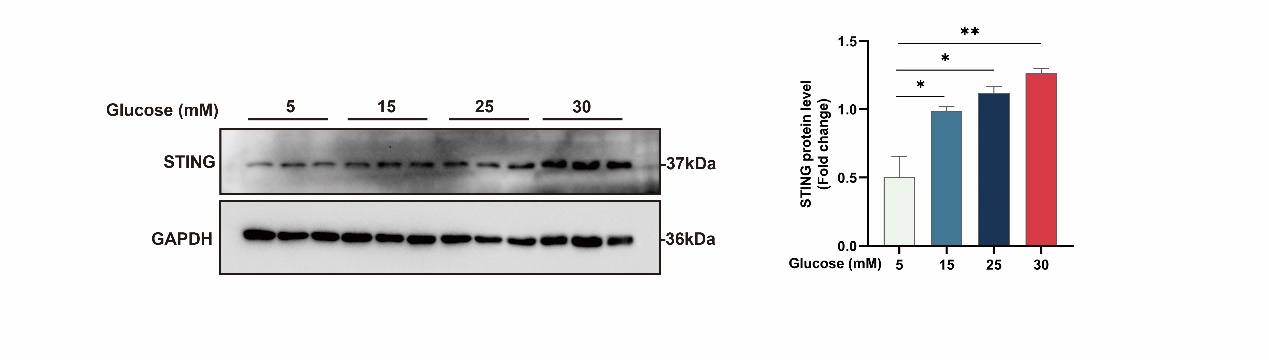


**Figure S3. Exposure of C2C12 myotubes to glucose increased the protein level of STING in a concentration-dependent manner**. Data were analyzed using unpaired two-tailed Student’s t-test. Data are presented as mean ± SEM. *p<0.05, **p<0.01.


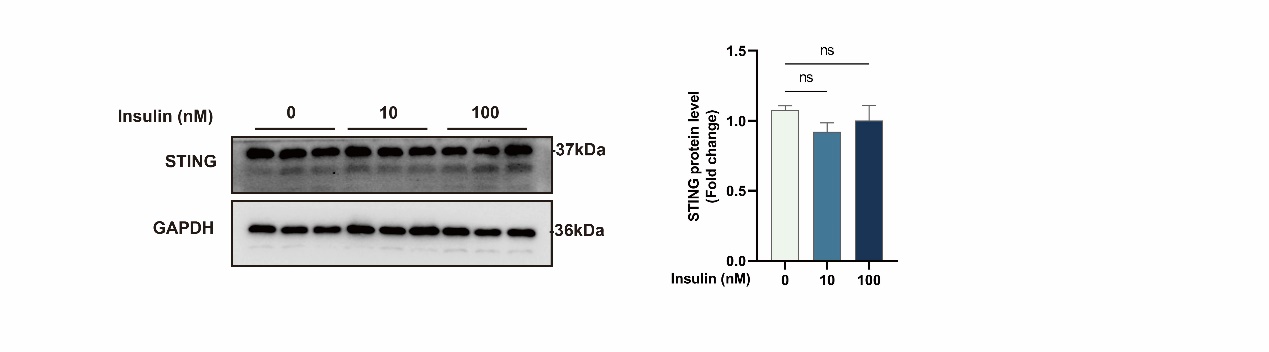


**Figure S4.** **Treatment of the C2C12 myotubes with insulin had no effect on the protein level of STING**. Data were analyzed using unpaired two-tailed Student’s t-test. Data are presented as mean ± SEM; ns, not significant.

**
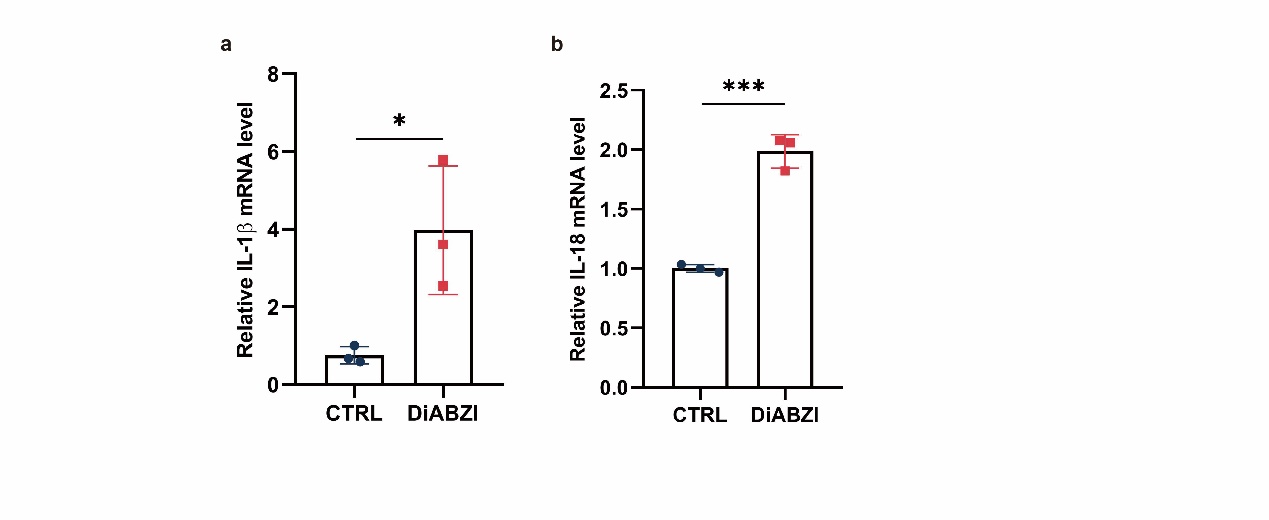
**

**Figure S5. Activating STING contributed to muscle atrophy and pyroptosis in mouse C2C12 myotubes**. Quantitative PCR analysis of mRNA levels of IL-1β and IL-18 (n=3). **Data were analyzed using unpaired two-tailed Student’s t-test.** Data are presented as mean ± SEM. *p<0.05, ***p<0.001.


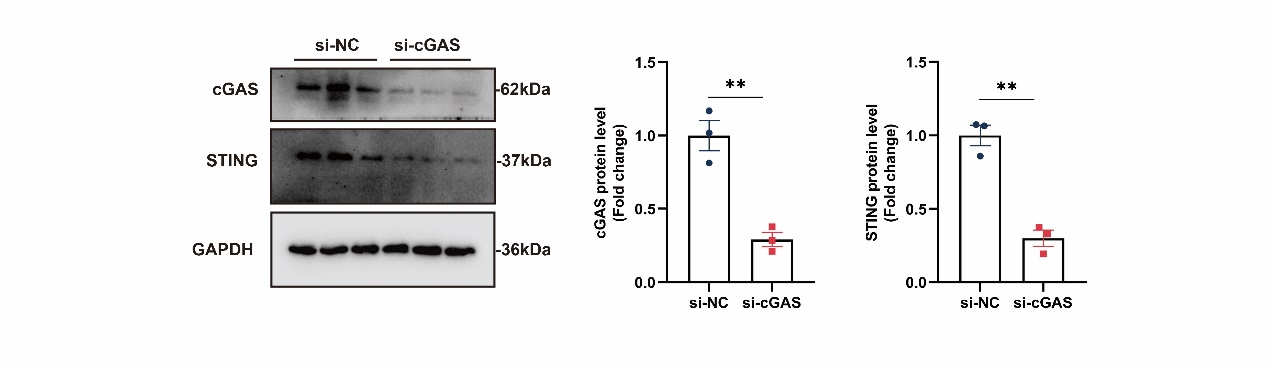


**Figure S6. Transfected with si-cGAS significantly downregulated STING expression in mouse C2C12 myotubes treated with glucose.** Data were analyzed using unpaired two-tailed Student’s t-test. Data are presented as mean ± SEM; **p<0.01.

**
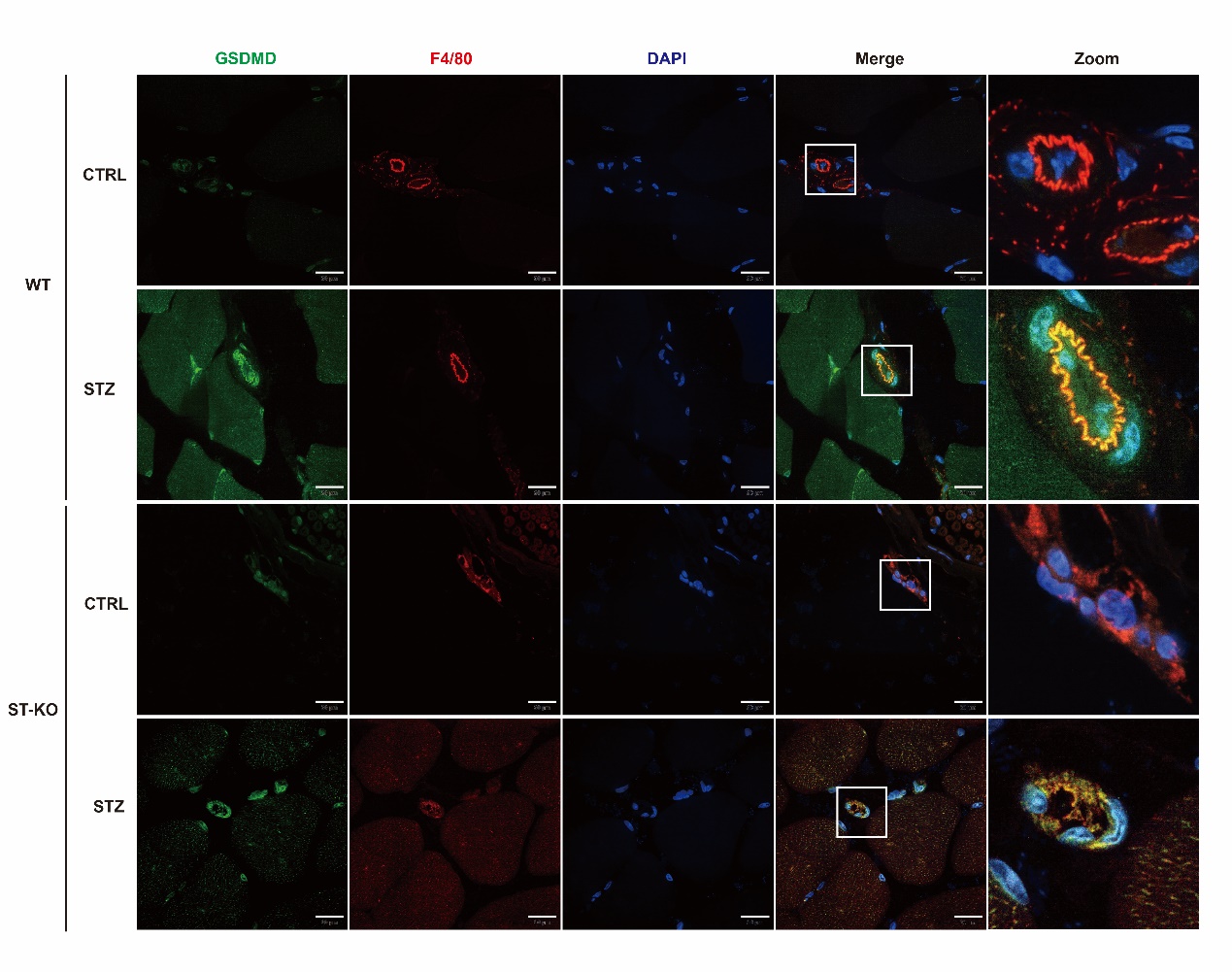
**

**Figure S7. STING deficient did not affect the macrophage pyroptosis in GM tissues.** Representative images of GSDMD and F4/80 (macrophage marker) in GM tissues. Scale bar, 20 μm. GM, gastrocnemius muscle; STZ, streptozotocin; WT, wild type; ST-KO, STING-KO.

##
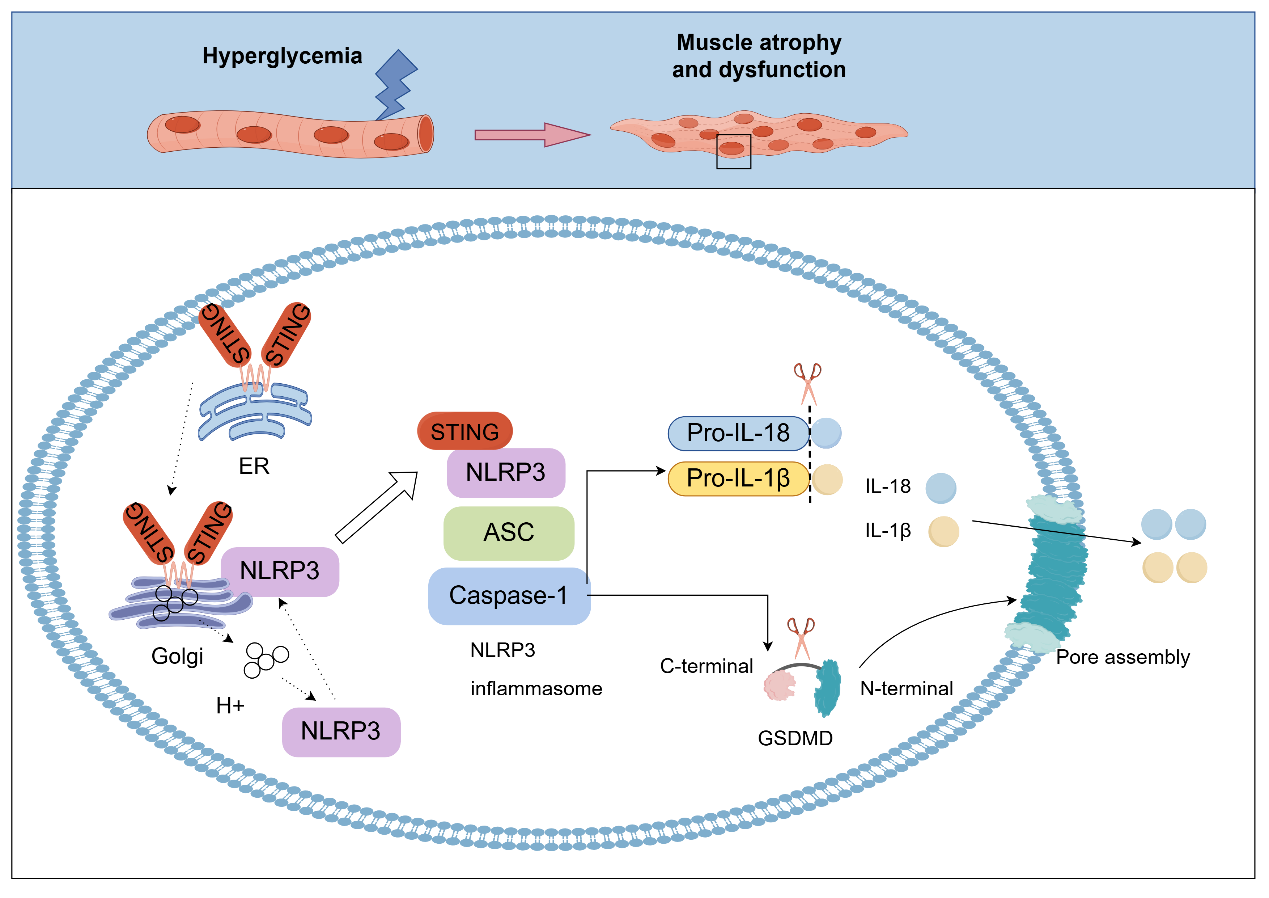


## Figure S8. A mechanism diagram. STING-induced activation of the NLRP3 inflammasome could lead to pyroptosis, resulting in muscle atrophy and dysfunction in diabetes. ER, endoplasmic reticulum.


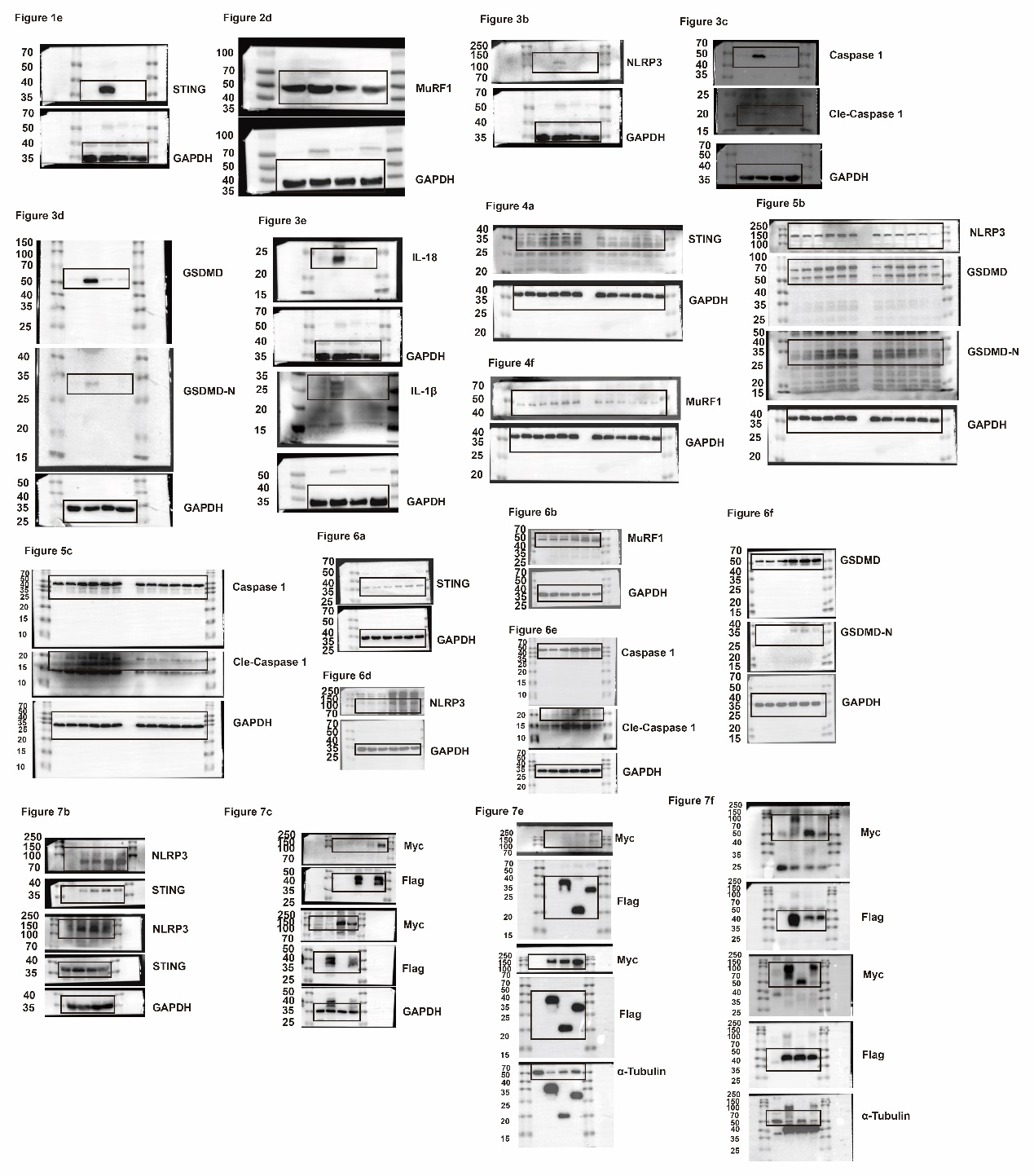


**Figure S9. Original blots for western blot assay in the present study.**

**Table S1 Primers used for qRT-PCR**

| **Gene name** | **Sequence (5’-3’)** |
| --- | --- |
| GAPDH | Forward: TGGCCTTCCGTGTTCCTAC |
|  | Reverse: GAGTTGCTGTTGAAGTCGCA |
| Murf1 | Forward: AGGACTCCTGCAGAGTGACCAA |
|  | Reverse: TTCTCGTCCAGGATGGCGTA |
| Atrogin | Forward: GCAAACACTGCCACATTCTCTC |
|  | Reverse: CTTGAGGGGAAAGTGAGACG |
| IL-18 | Forward: GTTTACAAGCATCCAGGCACA |
|  | Reverse: TCATGCAGCCTCGGGTATTC |
| IL-1β | Forward: GCAACTGTTCCTGAACTCAACT |
|  | Reverse: ATCTTTTGGGGTCCGTCAACT |
